# Nitroimidazole Action in *Entamoeba histolytica:* A Central Role for Thioredoxin Reductase

**DOI:** 10.1371/journal.pbio.0050211

**Published:** 2007-07-31

**Authors:** David Leitsch, Daniel Kolarich, Iain B. H Wilson, Friedrich Altmann, Michael Duchêne

**Affiliations:** 1 Department of Specific Prophylaxis and Tropical Medicine, Center for Physiology and Pathophysiology, Medical University of Vienna, Vienna, Austria; 2 Department of Chemistry, University of Natural Resources and Applied Life Sciences, Vienna, Austria; University of Vermont, United States of America

## Abstract

Metronidazole, a 5-nitroimidazole drug, has been the gold standard for several decades in the treatment of infections with microaerophilic protist parasites, including Entamoeba histolytica. For activation, the drug must be chemically reduced, but little is known about the targets of the active metabolites. Applying two-dimensional gel electrophoresis and mass spectrometry, we searched for protein targets in E. histolytica. Of all proteins visualized, only five were found to form adducts with metronidazole metabolites: thioredoxin, thioredoxin reductase, superoxide dismutase, purine nucleoside phosphorylase, and a previously unknown protein. Recombinant thioredoxin reductase carrying the modification displayed reduced enzymatic activity. In treated cells, essential non-protein thiols such as free cysteine were also affected by covalent adduct formation, their levels being drastically reduced. Accordingly, addition of cysteine allowed E. histolytica to survive in the presence of otherwise lethal metronidazole concentrations and reduced protein adduct formation. Finally, we discovered that thioredoxin reductase reduces metronidazole and other nitro compounds, suggesting a new model of metronidazole activation in E. histolytica with a central role for thioredoxin reductase. By reducing metronidazole, the enzyme renders itself and associated thiol-containing proteins vulnerable to adduct formation. Because thioredoxin reductase is a ubiquitous enzyme, similar processes could occur in other eukaryotic or prokaryotic organisms.

## Author Summary

The protist parasites *Entamoeba histolytica, Trichomonas vaginalis,* and Giardia intestinalis grow in environments with low oxygen concentration. Infections with these parasites are commonly treated with metronidazole, a nitroimidazole drug that must be reduced for activation, resulting in several toxic metabolites. We examined the soluble proteome of metronidazole-treated E. histolytica cells for target proteins of these metabolites, applying two-dimensional gel electrophoresis and mass spectrometry. Of about 1,500 proteins visualized, only five formed covalent adducts with metronidazole metabolites, including thioredoxin, thioredoxin reductase, and superoxide dismutase. Metronidazole-bound thioredoxin reductase displayed diminished activity. In addition to these proteins, small thiol molecules, including cysteine, formed adducts with metronidazole. Supplementation with cysteine allowed the cells to survive otherwise lethal metronidazole concentrations. Finally, we discovered that one of the modified proteins, thioredoxin reductase, reduces metronidazole, suggesting a central role for this enzyme with regard to metronidazole toxicity. Taken together, our work reveals a new area of molecular interactions of activated metronidazole with cellular components. Because thioredoxin reductase is a ubiquitous enzyme, similar processes could also occur in other eukaryotic or prokaryotic organisms.

## Abstract 3

Metronidazole is used for treatment of infections with microaerophilic protist parasites. Here, a new model of metronidazole activation is proposed, with a central role for thioredoxin reductase.

## Introduction

Entamoeba histolytica is a microaerophilic protozoan parasite and the causative agent of amoebiasis, a disease that affects millions of people worldwide and claims up to 100,000 casualties per annum [1]. As is the case with other microaerophilic parasitic infections, such as giardiasis (caused by Giardia intestinalis) and trichomoniasis (caused by Trichomonas vaginalis), the 5-nitroimidazole drug metronidazole has established itself as the most effective treatment of amoebiasis. Due to the high prevalence of these infections [2] and due to its role as a second-line defense against Helicobacter pylori infections [3], metronidazole has been included in the “essential medicines” list by the World Health Organization [4]. Metronidazole, like other nitroimidazoles, requires reduction at the nitro group in order to be transformed into its cytotoxic form, the nitroradical anion [5]. The activated nitro group undergoes further reduction so that a nitrosoimidazole is generated [6] which can react with sulfhydryl groups [7] and with DNA [8] while being further reduced to an amine via a hydroxylamine intermediate. In the presence of oxygen, however, the nitroradical anion is suggested to be rapidly reoxidized to its respective parent drug before nitroso intermediates can be formed, i.e., a redox cycling effect also termed “futile cycle” [9]. Despite the resulting oxidative stress, this futile cycle is believed to render metronidazole treatment safe in man. However, there are still concerns regarding its potential carcinogenicity [10].

Since reduction of the nitro group is essential for nitroimidazole toxicity, extensive research has been dedicated to enzymes that can act as metronidazole-activating nitroreductases. In rat liver extracts, the microsomal enzyme NADPH-cytochrome P450 reductase was found to be responsible for nitroimidazole activation [11]. The microaerophilic parasites *G. intestinalis, T. vaginalis,* and *E. histolytica,* however, lack mitochondria [12] but depend on substrate-level phosphorylation [13]. In these organisms, ferredoxin, which is being reduced by pyruvate:ferredoxin oxidoreductase (PFOR), has been suggested to activate metronidazole [14]. Indeed, in *T. vaginalis,* metronidazole activation was found to take place in the hydrogenosome [15], a hydrogen-producing organelle in which PFOR and ferredoxin are localized [16]. Moreover, purified ferredoxin was shown to be able to reduce various nitroimidazoles in vitro [17]; and in some highly metronidazole-resistant laboratory T. vaginalis strains, PFOR and ferredoxin were absent, stressing a direct relationship between ferredoxin and metronidazole activation in vivo [18]. Likewise, PFOR activity [19,20] and ferredoxin levels were reduced in metronidazole-resistant G. intestinalis strains [21]. In partially metronidazole-resistant *E. histolytica,* expression of ferredoxin 1 was sharply decreased [22] although PFOR levels remained unaltered [23]. In contrast to T. vaginalis [24], metronidazole resistance in E. histolytica could be mainly attributed to the increased expression of the antioxidant enzymes peroxiredoxin [22] and superoxide dismutase [23], rather than to loss of PFOR activity, as observed in the other two parasites.

For several decades, great efforts have been undertaken to deepen the understanding of metronidazole activation in the parasitic cell, but the exact mode of action in vivo of this pivotal drug has remained rather understudied. DNA is suggested to be the major target of metronidazole [14,25], as implied by several in vitro studies addressing metronidazole's mutagenicity and DNA-binding capability [8]. In addition, in vitro adduct formation of nitroimidazoles with proteins and thiols, e.g., cysteine, was also demonstrated [11], but specific targets in the treated parasites were never defined because nitroimidazole action was assumed to be indiscriminate. As a contribution to fill this gap, it was our goal to elucidate the processes that occur in the E. histolytica cell during metronidazole treatment at concentrations that are applied during the treatment of amoebiasis, and, if existent, identify specific targets of metronidazole. After completion of the E. histolytica genome project [26], application of proteomic methods such as two-dimensional gel electrophoresis (2DE) was greatly facilitated, permitting a comprehensive and rapid identification of proteins affected by metronidazole in treated E. histolytica cells.

In this study, we show that, in *E. histolytica,* activated metronidazole does not bind to protein indiscriminately, but reproducibly forms covalent adducts with a small and defined number of proteins, including enzymes such as thioredoxin reductase, superoxide dismutase, and purine nucleoside phosphorylase, as well as the multiple-role reductant protein thioredoxin. When recombinantly expressed in Escherichia coli BL21 (DE3) in the presence of metronidazole, the capability of thioredoxin reductase to reduce thioredoxin was significantly diminished. Moreover, levels of non-protein thiols, e.g., cysteine, were found to be drastically lowered in metronidazole-treated E. histolytica cells due to adduct formation between activated metronidazole and accessible sulfhydryl groups. In accordance with this finding, addition of cysteine to the growth medium allowed the cells to survive otherwise lethal metronidazole concentrations and significantly reduced protein adduct formation. Finally, we propose an alternative mode of metronidazole activation by thioredoxin reductase, because it showed nitroreductase activity in enzymatic assays.

## Results

### Metronidazole Modifies a Small Number of Proteins in E. histolytica

**Figure 1:**
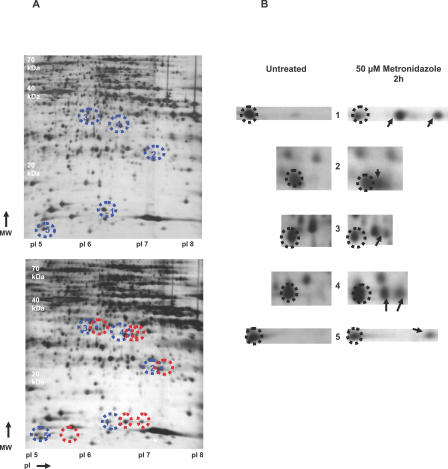
2DE of E. histolytica Cell Extracts Reveals Adduct Formation upon Metronidazole Treatment (A) The adducts formed can be identified as shifts to more basic pI values on 2D gels. Cells were treated with 50 μM metronidazole for 2 h or left untreated. Isoelectric focusing was performed between pH 5 and pH 8; SDS PAGE was run on 12.5% polyacrylamide gels. The proteins are marked on a section of the 2D gel of the untreated sample (upper gel image) and of the treated sample (lower gel image). Protein 1: metronidazole target protein 1 (two shifts); protein 2: superoxide dismutase (one shift); protein 3: purine nucleoside phosphorylase (one shift); protein 4: thioredoxin reductase (two shifts); and protein 5: thioredoxin (one shift). Unmodified proteins are marked by blue dotted circles, and shifted proteins are indicated by red circles. (B) The same proteins in a close-up: unmodified proteins are encircled, and the respective modified isoforms are indicated by arrows.

After having treated E. histolytica trophozoites for different time periods (1 h, 2 h, 3 h, and 6 h) and with varying metronidazole concentrations (10 μM–1 mM), cell lysates were prepared for 2DE experiments. Metronidazole concentrations between 50 μM and 100 μM proved to be the most suitable because cells were viable for more than 5 h, a time span which is sufficient for the cell to react to stress by expression of mRNA and proteins. In addition, therapeutic levels lie within this range. Because higher metronidazole concentrations led to rapid disintegration of the cells, and incubation periods for more than 2 h with 50 μM metronidazole did not reveal any additional changes in the protein profile, we chose exposure to 50 μM metronidazole for 2 h as our standard condition when challenging cells with metronidazole. We reproducibly found seven new protein spots on the gels that were isolated and analyzed by mass spectrometric tryptic peptide fingerprinting in combination with additional verification of selected peptide sequences by tandem mass spectrometry (MS/MS) (identified peptides and tandem mass spectra are listed in Figures S1–S5). These seven spots corresponded to five proteins (Figure 1A and 1B), identified as superoxide dismutase, purine nucleoside phosphorylase, thioredoxin, thioredoxin reductase, and a protein designated as “hypothetical protein XP_650662” in the E. histolytica protein database (Table 1). The last will further be referred to as “metronidazole target protein 1” (Mtp1), since its presence on 2D gels abolishes its hypothetical status. Surprisingly, the new spots did not correspond to newly synthesized protein, but appeared at the expense of other neighboring spots as shifted isoforms at a more basic isoelectric point (pI) (Figure 1A and 1B). The widths of the shifts in pI differed with each of the proteins. Thus, it was hypothesized that metronidazole exposure leads to modification of these five proteins, and that the newly appearing spots correspond to isoforms of pre-existing protein in the cell. Concomitant treatment of the cells with 100 μM cycloheximide to block protein synthesis did not prevent the appearance of the shifts on the gel when cells were exposed to metronidazole (unpublished data). This supported our notion that the observed spots do not correspond to newly synthesized protein. Moreover, we did not find any proteins significantly up-regulated or down-regulated in expression during metronidazole exposure; mRNA expression was also not found to be altered (M. Tazreiter, unpublished data). Thus, presumably, E. histolytica does not react to short-term metronidazole treatment by mRNA synthesis or by synthesis of proteins that are involved in stress response or antioxidant defense. This indicated that the protein shifts might be due to metronidazole adduct formation with the five proteins rather than to a general stress response of the cell.

**Table 1:**
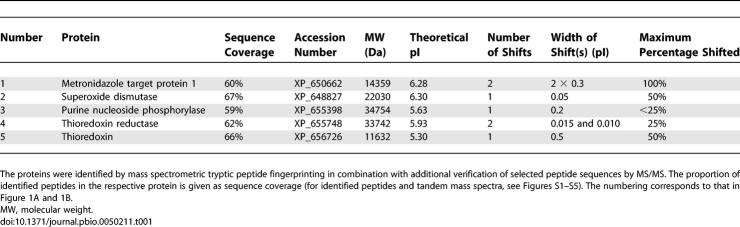
List of the Proteins Found to Be Shifted to Higher pI on 2D Gels upon Metronidazole Treatment of E. histolytica

**Figure 2:**
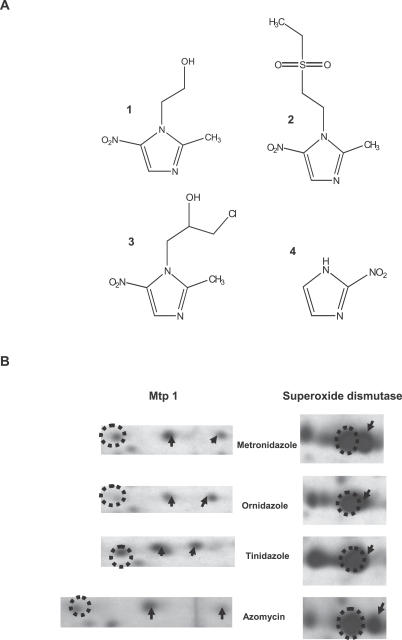
Different Nitroimidazoles Shift the Same Target Proteins to a Different Extent (A) The nitroimidazoles used in this study are shown: the 5-nitroimidazoles metronidazole (1), tinidazole (2), and ornidazole (3), and the 2-nitroimidazole azomycin (4). Different side chains result in different contributions of the compounds to the pI shifts on 2D gels. (B) Adduct formation of the same proteins with different nitroimidazoles. The same proteins were shifted with the 5-nitroimidazoles metronidazole, ornidazole, and tinidiazole, and with the 2-nitroimidazole azomycin. The width of the shifts in pI depended on the total charge of the nitroimidazole used, which is determined by its side chains. Shifts are exemplified by two of the proteins found: superoxide dismutase and metronidazole target protein 1. The unmodified proteins are encircled, and shifted proteins are indicated by arrows.

Unfortunately, mass spectrometric analysis of tryptic fragments did not reveal any metronidazole-bound peptides. Thus, in order to confirm metronidazole binding to the proteins, we treated the cells with other nitroimidazoles, including the 2-nitroimidazole azomycin, and the two 5-nitroimidazoles tinidazole and ornidazole (Figure 2A). Since cross-resistances were reported for most of the nitroimidazoles [2], we expected all nitroimidazoles to give similar results as compared to metronidazole. Again, treatment regimens with 50 μM of the respective nitroimidazole for 2 h were chosen as the experimental conditions. Indeed, two-dimensional (2D) gels revealed that the same proteins were affected, but the width of the pI shifts to the basic differed according to the varying pKa's of the nitroimidazoles, which can be attributed to the different side chains at the N1 position. Protein shifts upon ornidazole treatment were slightly narrower, whereas protein shifts with tinidazole were considerably narrower than those observed with metronidazole (Figure 2B). Interestingly, the 2-nitroimidazole azomycin also shifted the same proteins. With regard to pI interval, the shifts by azomycin were wider than shifts by the other nitroimidazoles tested, but the amount of the respective proteins shifted was smaller. The modification of proteins seemed to be independent of the position of the nitro group in the ring, indicating a generalized pattern of nitroimidazole action in E. histolytica.

Quantitative analysis with Melanie 2DE imaging software indicated that, even after prolonged incubation, only a defined fraction of each protein was shifted until a certain maximum was reached (Table 1). Thus, higher nitroimidazole concentrations only allowed this maximum to be more rapidly attained (unpublished data). The proteins were also modified to the same extent in partially metronidazole-resistant E. histolytica cells that had been continuously cultured in the presence of 10 μM metronidazole (unpublished data). Like others before [22,23], we were not able to obtain highly metronidazole-resistant E. histolytica strains. Such would have been very helpful for an assessment of the impact of the modifications on metronidazole-mediated toxicity.

### Metronidazole Reduces the Levels of Non-Protein Thiols in the Cell

**Figure 3:**
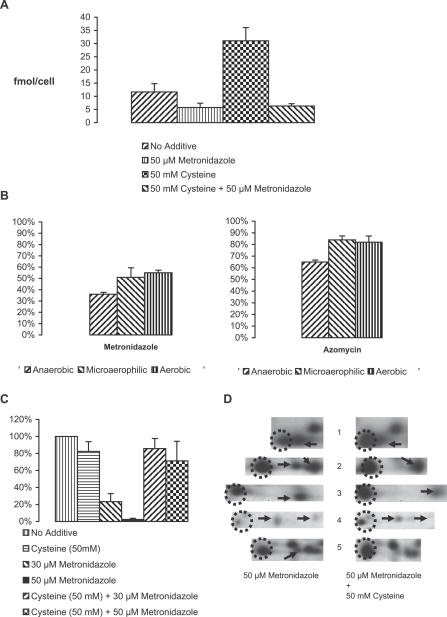
Interdependence of Non-Protein Thiols and Metronidazole Toxicity (A) Metronidazole diminishes non-protein thiol levels in the cell. Determination of total non-protein sulfhydryl groups (fmol/cell), i.e., free thiols, in the cell after treatment of E. histolytica cells for 2 h with either additional 50 mM cysteine (67 mM in total) or 50 μM metronidazole alone, or both in combination. The experiment was independently performed three times. Vertical bars indicate standard deviations. (B) The decrease of non-protein thiols is most pronounced under anaerobic conditions when treating cells with metronidazole and azomycin. Cells were either treated with 50 μM of metronidazole (left) or azomycin (right) for 2 h under anaerobic (0% O_2_ and 18% CO_2_), microaerophilic (5% O_2_ and 8% CO_2_), and aerobic (21% O_2_ and 0.4% CO_2_) conditions. Values are given as percentages of non-protein thiol concentrations of the respective untreated samples. (C) Cysteine counteracts metronidazole toxicity. E. histolytica cells were treated with either 30 μM or 50 μM of metronidazole in the presence or absence of additional 50 mM cysteine for 20 h. Viability of cells was determined by trypan blue exclusion. The experiment was independently repeated three times. Numbers of viable cells are given as percentages of total cell counts. Vertical bars indicate standard deviations. (D) Cysteine reduces adduct formation of proteins with metronidazole. Cells were treated with 50 μM of metronidazole either in the presence or absence of additional 50 mM cysteine for 2 h. Afterwards, cell lysates were prepared, and the extent of adduct formation of superoxide dismutase (1), thioredoxin reductase (2), thioredoxin (3), metronidazole target protein 1 (4), and purine nucleoside phosphorylase (5) with metronidazole was visualized by 2DE. Unmodified proteins are encircled, and shifted proteins are indicated by arrows.

Because nitroimidazoles have been found to form adducts with sulfhydryl group containing compounds [7], we tested whether metronidazole treatment could reduce the levels of non-protein thiols in the cell, e.g., cysteine, which constitutes the major reductant in E. histolytica [27,28]. Treatment with 50 μM metronidazole for 2 h decreased non-protein thiol levels to 49% (Figure 3A) of those in untreated cells (11 fmol/cell), whereas additional 50 mM cysteine (17 mM cysteine constitutes the standard concentration in TYI-S-33 medium), led to accumulation of cysteine in the cell and raised non-protein thiol levels by about 180%, to approximately 31 fmol/cell. When metronidazole and cysteine were used in combination, thiol levels were also sharply decreased (54% of the untreated state). Since cysteine could not accumulate intracellularly in the presence of metronidazole (the observed 5% difference between total free-thiol levels of cells treated with metronidazole alone and those treated with metronidazole in combination with cysteine, is statistically not significant), these results indicate that cysteine levels in the cell are diminished by metronidazole.

As the observed decrease in non-protein thiol levels could also be the consequence of oxidative stress, e.g., oxidation of sulfhydryl groups by hydrogen peroxide, we attempted to verify covalent adduct formation of metronidazole with sulfhydryl groups by exposing the cells to 50 μM metronidazole under anaerobic (Merck Anaerocult A, 0% O_2_ and 18% CO_2_), microaerophilic (Merck Anaerocult C, 5% O_2_ and 8% CO_2_), and aerobic conditions (aerated vials), respectively. We reasoned that, if the decrease in thiol levels were to be attributed to oxidative stress, exposure of the cells to metronidazole under aerobic conditions would result in even more strongly reduced thiol levels, whereas, under anaerobic conditions, the observed effect should be significantly mitigated or even absent. However, our experiment showed that the reduction of thiol levels, to 36% of the level of untreated cells, was the most strongly pronounced after metronidazole exposure under anaerobic conditions (Figure 3B), followed by exposure to metronidazole under microaerophilic conditions, with 51%, and then under aerobic conditions, with 54%. Thus, oxygen did not promote, but conversely, counteracted the reduction of thiol levels upon metronidazole exposure, which strongly suggests that covalent adduct formation of activated metronidazole with thiol groups is the reason for the decrease in non-protein thiol levels. Moreover, these results are in line with the finding that oxygen can detoxify nitroradical anions and regenerate the parent drug by snatching the electron from the nitro group [9]. However, this effect was not as pronounced as anticipated, because reoxidation of the metronidazole radical anion by oxygen was incomplete, leading to almost halved non-protein thiol levels, even under aerobic conditions. Interestingly, adduct formation with the five proteins found was not affected in the presence of higher oxygen concentrations when cells were treated with 50 μM metronidazole for 2 h (unpublished data).

### Azomycin Also Diminishes Non-Protein Thiols but to a Lesser Extent

In order to evaluate the extent to which the observed decrease in non-protein thiol levels contributes to metronidazole toxicity, we treated cells with azomycin, which had been found to be by far less toxic to E. histolytica than metronidazole (D. Leitsch, unpublished data). Indeed, non-protein thiol levels were found to be affected by azomycin, even if considerably less than by metronidazole, paralleling the observation that azomycin shifts the same proteins as metronidazole, albeit to a smaller extent. Under anaerobic conditions, a drop to 65% of the original level when treating cells with 50 μM azomycin for 2 h, as compared to a drop to 36% when treating the cells with metronidazole, was observed (Figure 3B). In the presence of oxygen, non-protein thiol levels after exposure to azomycin were only slightly reduced: 84% (microaerophilic) and 82% (aerobic), respectively, of the non-protein thiol levels were retained as compared to untreated cells. Interestingly, higher oxygen levels counteracted adduct formation of azomycin with non-protein thiol groups more strongly than was the case with metronidazole. As compared to anaerobic conditions, 49% less adduct formation with azomycin (a drop of non-protein thiols of only 18%—to 82% of the untreated sample—instead of a drop of 35%—to 65%) and 28% less adduct formation with metronidazole (a drop of 46%—to 54% of the untreated sample—instead of a drop of 64%—to 36%) were observed when treating cells under aerophilic conditions. Differences between microaerophilic and anaerobic conditions with regard to their impact on the decrease of non-protein thiol levels in the presence of metronidazole or azomycin were hardly significant, if existent.

### Cysteine Confers Protection against Metronidazole and Reduces Adduct Formation of Metronidazole with Proteins

Although non-protein thiol levels were only insignificantly higher in cells that were treated with metronidazole and cysteine in combination, as compared to non-protein thiol levels in cells treated with metronidazole alone, we observed that the cells were not rounding off and disintegrating in the presence of higher cysteine levels. Therefore, we tested whether raised cysteine levels in the medium could protect E. histolytica during metronidazole treatment. Cells were treated with 30 μM or 50 μM metronidazole either in the presence or absence of additional 50 mM cysteine (67 mM in total) (Figure 3C). Addition of 50 mM cysteine slightly impaired viability; only 83% of the cells were still viable after 20 h of incubation. In the cultures treated with 30 μM metronidazole alone, only 23% of the cells were still viable after the same time span, whereas 50 μM of metronidazole was sufficient to kill almost all cells in the culture (2% viable cells). However, 86% of the cells in the culture treated with 30 μM metronidazole and 71% of the cells treated with 50 μM metronidazole were still viable after 20 h when 67 mM cysteine was present in the growth medium. These results clearly indicate that cysteine strongly counteracts metronidazole toxicity in E. histolytica.

We speculated that the protective effect of cysteine might be due to less metronidazole adduct formation with proteins, because we expected free cysteine to compete with proteins for activated metronidazole. Thus, we assessed the influence of raised cysteine levels on adduct formation of metronidazole with the five proteins identified. Additional 50 mM cysteine markedly reduced metronidazole adduct formation with protein when cells were treated with 50 μM metronidazole for 2 h (Figure 3D). The shifts in pI of superoxide dismutase, thioredoxin, and Mtp1 were clearly diminished in this case, whereas thioredoxin reductase was only subject to putatively one modification and purine nucleoside phosphorylase remained unmodified. The latter two observations were not always made when repeating the experiment, but a very distinct decrease of adduct formation with all five proteins was perfectly reproducible. These results suggest that non-protein cysteine competes with proteins in the formation of adducts with activated metronidazole, and that the observed protective effect of cysteine during metronidazole exposure might be due to fewer protein adducts formed.

### Expression of Recombinant E. histolytica Thioredoxin Reductase and E. histolytica Thioredoxin

As a result of our observation that metronidazole adducts are also formed in *Es. coli* under microaerophilic conditions, albeit at much higher metronidazole concentrations than is the case in E. histolytica (unpublished data), we decided to recombinantly express *E. histolytica* thioredoxin reductase and E. histolytica thioredoxin in *Es. coli* either in the presence or in the absence of metronidazole. We speculated that this strategy would allow us to obtain a large quantity of metronidazole-bound protein that could aid in the mass spectrometric identification of the observed modifications. In addition, the influence of metronidazole adduct formation on protein function could be assayed in vitro with the modified and unmodified recombinant proteins at hand. As thioredoxin and thioredoxin reductase are of central importance for the cell's physiology [29], these two proteins were of particular interest to us. Moreover, human [30] and Arabidopsis thaliana [31] thioredoxin reductases were shown, in addition to their intrinsic disulfide reductase activity, to reduce nitro compounds such as tetryl or 1-chloro-2,4-dinitrobenzene (CDNB). Therefore, we also wanted to determine in vitro whether E. histolytica thioredoxin reductase can, in addition to its role as a disulfide reductase, reduce nitroimidazoles.

Recombinant E. histolytica thioredoxin reductase (recEh TrxR) and recombinant E. histolytica thioredoxin (recEh Trx) were produced in *Es. coli* BL21 (DE3) cells and purified on Ni-NTA columns via their carboxy-terminal hexahistidine tags. RecEh TrxR had a deep yellowish color that can be attributed to its FAD or FMN cofactor [32].

### Detection of Metronidazole Adducts on recEh TrxR and recEh Trx after Expression in the Presence of Metronidazole

**Figure 4:**
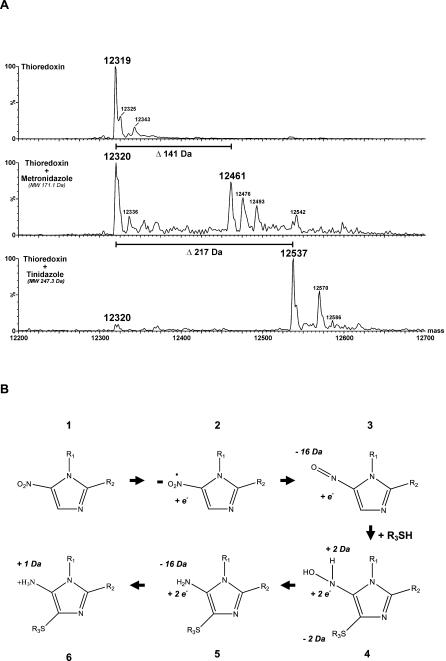
RecEh Trx Increases in Mass upon Modification with Metronidazole or Tinidazole (A) Detection of mass increment by LC-ESI-QTOF-MS. Total protein mass was determined using LC-ESI-QTOF-MS after His-Tag purification of the protein. When BL21 (DE3) cells were not exposed to metronidazole or tinidazole during expression of recEh Trx, a single peak in the deconvoluted mass spectrum was observed. This peak was in good agreement with the expected mass of 12,322 Da when taking into account that methionine at position 1 is removed, six histidines are attached at the C-terminus, and one cysteine-disulfide bridge is formed. When BL21 (DE3) cells were exposed to metronidazole or tinidazole during recombinant protein expression, recEh Trx modified with metronidazole or tinidazole eluted slightly earlier on the LC than unmodified recEh Trx. The determined mass increments, i.e., 141 Da with metronidazole treatment and 217 Da with tinidazole, support the reaction scheme for 5-nitroimidazoles as proposed by Wislocki and colleagues [33]. Additional peaks in the spectra of modified recEh Trx correspond to mass increments of approximately 16 Da or 32 Da, and can be attributed to bound oxygen (a single oxygen or two). (B) Model of adduct formation of 5-nitroimidazoles with sulfhydryl groups. After the uptake of 5-nitroimidazoles into the cell (1), the nitro group is reduced to form a nitro radical anion (2). According to the model of Wislocki and colleagues [33], the nitro radical anion is further reduced (−16 Da) to a highly reactive 5-nitrosoimidazole (3) which forms adducts (−2 Da) with sulfhydryl groups via the C4 of the imidazole ring (4). Adduct formation is accompanied by reduction (2 e^−^) of the nitroso group to a hydroxylamine group (+2 Da). Subsequently (5), the hydroxylamine group is further reduced (2 e^−^) to an amino group (−16 Da), resulting in the stable and non-reactive 5-aminoimidazole adduct. At physiological pH or under the conditions applied during MS, the amino group is protonated (+1 Da) (6). A total of six e^−^ is necessary at the nitrogen of the former nitro group to obtain the aminoimidazole adduct which, compared to the corresponding nitroimidazole, decreases in mass by 30 Da. The bound protein or thiol loses one proton during adduct formation. Thus, in total, nitroimidazole adducts gain the molecular mass of the respective nitroimidazole less 31 Da.

When *Es. coli* BL21 (DE3) cells were treated with 1 mM metronidazole during recEh TrxR and recEh Trx expression, the recombinant proteins were efficiently modified by metronidazole as verified by 2DE (unpublished data). In contrast to our failed attempts to identify metronidazole on peptides that were directly isolated from 2D gels, we observed, by liquid chromatography electrospray ionization quadrupole time-of-flight tandem mass spectrometry (LC-ESI-QTOF-MS) of intact proteins, that a high proportion of recEh TrxR and recEh Trx displayed a shift in molecular mass (Figure 4A). The shift in the deconvoluted spectra of both recEh Trx and recEh TrxR (but here only illustrated with recEh Trx), corresponded to a mass gain of 141 Da. This is in good agreement with the in vitro reaction scheme for 5-nitroimidazoles as proposed by Wislocki and colleagues [33] (Figure 4B). In this scheme, the activated 5-nitroimidazole is first reduced to an electrophilic nitrosoimidazole, which is subsequently attacked at its C4 atom by a sulfhydryl group. This is accompanied by further reduction to a hydroxylamine group, which is finally reduced to an amino group. Thus, the nitroimidazole loses two oxygen atoms from the nitro group and one proton from C4, and gains two hydrogens, resulting in a decrease in mass of 31 Da. At physiological pH and under the conditions applied during LC-ESI-QTOF-MS, however, the amino group is protonated, leading to a total mass decrease of only 30 Da. Since metronidazole has a mass of 171.16 Da, a protein that binds metronidazole can be expected to increase in mass by about 140 Da because it gains 141 Da from the bound 5-aminoimidazole derivate of metronidazole but loses the proton of a sulfhydryl group.

When allowing for the methodological restraints of the LC-ESI-QTOF-MS instrument, which has a mass deviation of ±4 Da when analyzing a protein of the size of recEH Trx, the calculated theoretical mass gain of 140 Da after modification by activated metronidazole corresponds well with the observed shift of 141 Da. In order to check whether the proposed model also applies for other 5-nitroimidazoles than metronidazole, we expressed recEh TrxR and recEh Trx in presence of tinidazole, which has a molecular mass of 247.3 Da. The result obtained with metronidazole was paralleled by that with tinidazole (Figure 4A); the shifts in the deconvoluted spectra amounted to 217 Da (molecular weight of the nitroimidazole less 30 Da). Moreover, it is important to add that the proposed model for nitroimidazole binding is also supported by the fact that, on 2D gels, the pI values of all five proteins identified were shifted to the basic, probably due to the reduction of the nitro group of the nitroimidazoles to a basic amino group.

Additional peaks that can be observed on the mass spectra of metronidazole- and tinidazole-bound recEh Trx correspond to mass increments of approximately 16 Da or 32 Da, and very likely can be attributed to oxidation (one or two oxygen atoms, respectively). Not unexpectedly, reactive oxygen species that are generated during nitroimidazole treatment led to oxidation of proteins (e.g., recEh Trx) and, probably, other cell constituents.

Unfortunately, our attempts so far to pinpoint the modifications to specific tryptic peptide fragments from recEh Trx and recEh TrxR have been as unsuccessful as had been our attempts to identify the modifications on E. histolytica proteins directly isolated from 2D gels. Obviously, the nitroimidazole adducts were not stable under the experimental conditions applied, possibly because incubation periods with trypsin (overnight at 37 °C) were too long or the conditions during LC-ESI-QTOF-MS were too harsh. We will, therefore, intensify our efforts in the future and modify the standard protocols accordingly.

### Metronidazole-Modified recEh TrxR Displays Considerably Reduced Thioredoxin Reductase Activity

**Figure 5:**
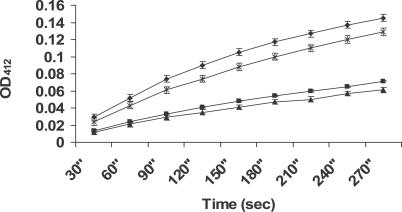
Metronidazole Binding Diminishes Thioredoxin Reductase Activity of recEh TrxR After expression of recEH TrxR and recEH Trx in the presence or absence of metronidazole for 3 h, proteins were isolated and thioredoxin reductase activity of recEh TrxR was determined by reduction of DTNB via recEh Trx as measured at λ = 412 nm (OD_412_). Diamonds (♦) indicate the use of unmodified forms of both recEh TrxR and recEh Trx, crosses (**x**) the use of unmodified recEH TrxR and metronidazole-modified recEh Trx. Squares () indicate the use of metronidazole-modified recEh TrxR and unmodified recEh Trx, and triangles (▴) the use of modified forms of both recEh TrxR and recEh Trx.

Thioredoxin reductase activity of recEh TrxR (applied at a concentration of 148 nM) was verified (Figure 5) by reduction of the disulfide 5,5′-dithiobis-(2-nitrobenzoic acid) (DTNB) to 2-nitro-5-thiobenzoic acid (TNB) via recEh Trx (applied at a concentration of 174 nM). Specific reduction of recEh Trx was determined by subtracting the ground-level reduction of DTNB (206 nmol min^−1^ mg^−1^) by recEh TrxR and was found to amount to 559 nmol min^−1^ mg ^−1^ (which equals a turnover of approximately 23.5 min^−1^). RecEh TrxR and its substrate, recEh Trx, were used in roughly equimolar amounts because our 2D gels suggested that both proteins are about equally abundant in the cell. Because we wanted to stay as close to physiological conditions as possible, we did not apply such a high excess of the substrate to the enzyme as would be necessary for the exact determination of the kinetic constants of recEh TrxR. Nevertheless, the activity of recEh TrxR, determined by us, is in good accordance with the thioredoxin reducing activity of E. histolytica thioredoxin reductase as determined just recently by Arias and colleagues [34].

We used metronidazole-modified recEh TrxR and recEh Trx for an estimation of the influence of metronidazole on protein function. Thioredoxin reducing activity of metronidazole-modified recEh TrxR dropped by more than 50% to 265 nmol min^−1^ mg^−1^ (Figure 5), and when used in combination with metronidazole-modified recEh Trx, the efficiency of recEh Trx reduction by recEh TrxR was even further diminished (220 nmol min^−1^ mg^−1^). When assaying metronidazole-modified recEh Trx alone, disulfide reduction lay also clearly below (427 nmol min^−1^ mg^−1^) the reduction rate as compared to using unmodified forms of both recEh TrxR and recEh Trx (559 nmol min^−1^ mg^−1^).

### RecEh TrxR Displays Nitroreductase Activity In Vitro

Using a recEH TrxR concentration of 118 nM (equal to 4 μg/ml), CDNB reduction was determined by measuring NADPH consumption at 340 nm (Table 2). Nitroreductase activity amounted to 233 nmol min^−1^ mg^−1^ (7.8 reduction events per minute per molecule of recEh TrxR) when using CDNB as the substrate at a concentration of 100 μM. Unfortunately, the assay was heavily disturbed by the absorbances of the nitroimidazoles. As an alternative method (Table 2), nitroreductase activity of recEh TrxR was indirectly determined by measuring reduction of cytochrome c by reduced nitro compounds [30], or by superoxide radical anions generated by the transfer of an electron from nitroradical anions to oxygen, respectively. In both cases, reduction of cytochrome c could be attributed to the previous reduction of the assayed nitro compounds, i.e., CDNB, the 2-nitroimidazole azomycin, and the 5-nitroimidazole metronidazole, by recEh TrxR (4 μg/ml). As expected, CDNB was readily reduced at a rate of 171 nmol^−1^ mg^−1^ at concentrations as low as 10 μM (Table 2). This rate lies in the range of the determined nitroreductase activity of recEh TrxR when measuring NADPH consumption. The 36% higher reduction of CDNB in the first assay is likely due to the 10-fold higher CDNB concentration used. The 2-nitroimidazole azomycin was also readily reduced at a concentration of 100 μM (63 nmol min^−1^ mg^−1^). The reduction rate of metronidazole (1 mM) amounted to 31 nmol min^−1^ mg^−1^. All values given above have been corrected for the ground-level activity of recEh TrxR, i.e., the reduction of molecular oxygen in the assay buffer, resulting in the formation of superoxide radical anions that, in turn, can reduce cytochrome c. In the absence of recEh TrxR, no reduction of cytochrome c was observed. In contrast to thioredoxin reductase activity, nitroreductase activity was not impaired with metronidazole-bound recEh Trx (unpublished data). This is possibly due to the fact that the flavin cofactor rather than the enzymatic site of recEh TrxR is responsible for nitroreduction [31].

**Table 2:**
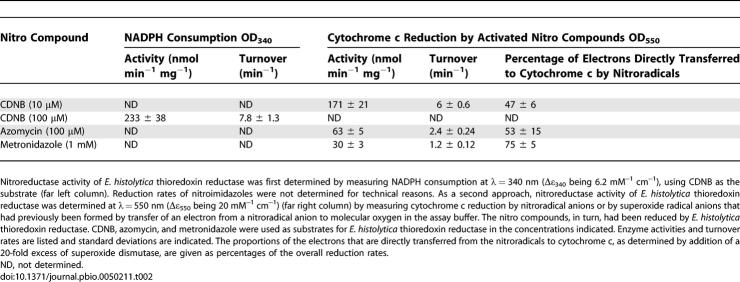
Nitroreductase Activity of RecEh TrxR

In order to distinguish between direct reduction of cytochrome c by nitroradical anions and between reduction of cytochrome c by superoxide radical anions that had previously been formed by the transfer of the electron of the nitroradical anion to molecular oxygen, superoxide dismutase was added to the reactions in about 20-fold excess (2.5 μM) (Table 2). The addition of superoxide dismutase completely abolished ground-level reduction of cytochrome c by EhTrxR and decreased cytochrome c reduction in the presence of 10 μM CDNB to 47% of the original value. Cytochrome c reduction in the presence of 100 μM azomycin was diminished to 53%, whereas 75% of the original value was retained with 1 mM metronidazole. Higher concentrations of superoxide dismutase did not lead to further decreases in cytochrome c reduction. Thus, in the case of CDNB and azomycin, roughly half of the cytochrome c reduction is mediated by superoxide radical anions, whereas the metronidazole nitroradical anions directly transferred most of the electrons to cytochrome c. These results provide direct evidence for the formation of superoxide radical anions upon nitroimidazole treatment in general and within limits upon metronidazole treatment, and thereby for the generation of oxidative stress in the microaerophilic cell.

## Discussion

In this study, we show for the first time that metronidazole forms adducts with proteins and non-protein thiols in an in vivo model, i.e., a parasite that is commonly treated with this drug. The shifts in masses, found on the mass spectra of metronidazole-bound recEh Trx and recEh TrxR, confirmed the in vitro model for 5-nitroimidazole adduct formation by Wislocki and colleagues [33] (Figure 4B). In contrast to the in vitro data from the late 1970s and early 1980s, however, we found discrete changes in the protein profile of E. histolytica after metronidazole treatment, i.e., modification of five proteins, rather than indiscriminate protein adduct formation. It is possible that there are some more proteins affected, because 2DE does not cover the whole proteome of a given organism. Very large proteins, low-abundance proteins such as transcription factors, and highly hydrophobic membrane proteins that could also potentially form adducts with nitroimidazoles cannot be identified by our approach. Nevertheless, even if allowing for these restraints, the number of affected proteins can be expected to remain small.

Because we made similar observations with Entamoeba dispar (the nonpathogenic relative of *E. histolytica)*, *G. intestinalis, T. vaginalis,* and *Es. coli* (unpublished data), we suggest that adduct formation with a defined subset of proteins can take place in any organism that is treated with nitroimidazoles (unpublished data): the presumed reduction of the nitro group to an amino group during adduct formation with the proteins leads to easily discernable shifts on 2D gels to more basic pI values. It is, therefore, interesting to speculate that nitroimidazoles could be an invaluable tool in proteomics, because our data suggest that they allow identification of nitroreductases and associated proteins by shifting them to more basic pI values that can easily be detected on 2D gels.

Apart from forming covalent adducts with proteins, metronidazole also diminishes non-protein thiol levels in the cell (Figure 3A), including that of cysteine. The observed decrease in non-protein thiol levels is due to covalent adduct formation with metronidazole and not due to oxidative stress generated by the activated drug, because the drop in thiol levels was most pronounced in the total absence of oxygen and least pronounced under aerobic conditions (Figure 3B).

Since cysteine is a compound of essential importance to E. histolytica cell physiology [35], and because it is assumed to function as the major reductant in the cell [27], its depletion could contribute to metronidazole toxicity in E. histolytica. On the other hand, cysteine could also predominantly have a protective role because other essential thiols in the cell, such as coenzyme A, might also form adducts with metronidazole. Interestingly, after 2 h of incubation, non-protein thiol levels in cells treated with 50 μM metronidazole and 50 mM cysteine were almost diminished to the same extent as non-protein thiol levels in cells treated with metronidazole alone (Figure 3A). Longer incubation periods with additional 50 mM cysteine in the growth medium, however, might lead to consumption and, consequently, detoxification of metronidazole. This is indicated by the observation that the toxic effect of metronidazole was drastically reduced after addition of 50 mM cysteine, because more than 70% of the cells in a culture survived 20-h exposure to (otherwise lethal) 50 μM metronidazole (Figure 3C). In addition, raised cysteine levels also led to less protein adduct formation after metronidazole treatment (Figure 3D), which indicates the interdependence of non-protein and protein sulfhydryl groups with regard to metronidazole toxicity and which could be an explanation for the protective effect of cysteine during metronidazole exposure.

Treatment of E. histolytica cells with the clearly less toxic 2-nitroimidazole azomycin also led to the modification of the five proteins found (Figure 2B) and a decrease in non-protein thiol levels (Figure 3B), albeit to a far smaller extent. Interestingly, the capability of azomycin to diminish non-protein thiol levels in the cell was also much more decreased by oxygen than was the case with metronidazole. Thus, the lower toxicity of azomycin could be based on its reduced tendency to form adducts with sulfhydryl groups and on its higher reactivity with oxygen (Table 2).

A potentially detrimental effect of metronidazole binding to protein function is indicated by the significant decrease of the thioredoxin reductase activity of recEh TrxR after metronidazole treatment (Figure 5). However, it is also conceivable that the observed oxidation of several amino acids concomitant with nitroimidazole binding, as observed with recEh Trx (Figure 4A), contributes to a diminished enzymatic function.

E. histolytica thioredoxin reductase was found to be a nitroreductase that is able in vitro to reduce CDNB, as well as the nitroimidazoles azomycin and metronidazole (Table 2). In contrast to thioredoxin reductase activity of recEh TrxR, nitroreductase activity of metronidazole-bound recEh TrxR was not decreased (unpublished data), suggesting that nitroreduction is directly exerted by the FAD or FMN cofactor [31,32]. Azomycin (2.4 min^−1^ at a concentration of 100 μM) was more effectively reduced in the nitroreductase assay than metronidazole (1.2 min^−1^ at a concentration of 1 mM), possibly due to the higher redox potential of azomycin (*E* ^1^_7_ = −418 mV) as compared to metronidazole (*E* ^1^_7_ = −486 mV) [36]. Interestingly, CDNB, which was the compound tested to be most efficiently reduced (6 min^−1^ at a concentration of 10 μM) by recEh TrxR, exerted only a mildly toxic effect on E. histolytica in our experimental setting—arguably because it does not form adducts with the same proteins as observed with nitroimidazoles and because it does not lead to a reduction of non-protein thiol levels in the cell (D, Leitsch, unpublished data). It is also possible, however, that CDNB does not enter the cell as readily as metronidazole.

When superoxide dismutase was added to the reactions, cytochrome c reduction rates in the presence of CDNB and azomycin were approximately halved (47% and 53%, respectively), whereas in the presence of metronidazole, 75% of the original rate was retained. These findings provide direct evidence for the generation of oxidative stress upon nitroimidazole treatment, because superoxide radical formation is evident. However, reoxidation of metronidazole by oxygen, with only 25% of the metronidazole nitroradical anions reoxidized, was by no means as complete and as rapid as anticipated [9]. Thus, it is doubtful whether the futile-cycle effect is really as influential on metronidazole toxicity as has been suggested.

Although the nitroreductase activity of recEH TrxR is rather low, it is comparable to the nitroreductase activities determined for A. thaliana and mammalian thioredoxin reductases [30,31]. According to our quantitative evaluations of 2D gels from E. histolytica cell extracts, thioredoxin reductase amounts to approximately 0.2% of the total protein in the cell, equaling 10–20 million copies per cell. Our estimate of the concentration of non-protein sulfhydryl groups amounts to 11 fmol/cell, i.e., only a 400-fold excess of non-protein sulfhydryl group levels over those of thioredoxin reductase. It is therefore conceivable that nitroimidazole reduction by thioredoxin reductase plays an important role in the decrease of non-protein thiol concentrations in the treated cell. In this context, it is interesting to note that studies in G. intestinalis have shown that the turnover of metronidazole reduction by purified ferredoxin was also not very pronounced (4 min^−1^), when purified PFOR instead of cell extract was used as the electron donor for ferredoxin [20].

We suggest that, due to spatial proximity to the reactive nitroimidazole species generated, reduction of nitroimidazoles by thioredoxin reductase renders this enzyme vulnerable to nitroimidazole adduct formation. Thioredoxin, superoxide dismutase, and Mtp1, in turn, can be expected to be localized in proximity to thioredoxin reductase or to interact with thioredoxin reductase. This could render these proteins prone to nitroimidazole modification as well. Proteins that do not interact with thioredoxin reductase are likely to be less affected, because activated nitroimidazoles react with non-protein thiols or other compounds before they can react with proteins that are more distant to the site of nitroimidazole reduction. Thioredoxin needs to be reduced by thioredoxin reductase in order to fulfill its multiple purpose as a reductant protein [29], whereas superoxide dismutase could be required to remove superoxide anion radicals that are indirectly generated by the nitroreductase activity of thioredoxin reductase. Superoxide dismutase might minimize the damage caused by superoxide when being positioned near thioredoxin reductase. Metronidazole target protein 1 (Mtp1) has no close homolog in any other organism whose genome has been sequenced so far, but it contains an O-glycosyl hydrolase domain and displays extended homology to an α-amylase in E. histolytica. Recent research suggests that thioredoxins are also of decisive importance for starch degradation in plants. Very strong evidence has been presented for thioredoxin-mediated regulation of α-amylases in barley grain [37]. Reduction of intramolecular disulfide bonds in amylases renders these enzymes more soluble, which is a prerequisite for amylase activity. Since Mtp1, as a protein of about 14 kDa, contains as many as six cysteines, it is conceivable that it requires thioredoxin in order to be functional. In the case of purine nucleoside phosphorylase, the situation could be different because arsenate reductase activity has been observed with human purine nucleoside phosphorylase [38]. This gives reason to speculate about the potential nitroreductase activity of the corresponding enzyme in E. histolytica. Possibly, purine nucleoside phosphorylase binds to the imidazole ring of nitroimidazole compounds,because purines have an imidazole moiety. However, in the same study, the authors found arsenate reductase activity to strongly depend on reductants, especially DTT, which suggests that purine nucleoside phosphorylase could require reduction by thioredoxin as well. Very surprisingly, we did not find PFOR or ferredoxin among the proteins forming adducts with metronidazole, although PFOR can be readily found on 2D gels when analyzing E. histolytica cell extracts [39]. High percentage (20%) acrylamide gels did not show any shifted proteins in the range of ferredoxin (approximately 6 kDa). It has been reported that, in contrast to T. vaginalis or *G. intestinalis,* PFOR was not found to be down-regulated in metronidazole-resistant E. histolytica [22,23], which supports the assumption that PFOR might not be involved in nitroimidazole activation in this organism. However, this is questioned by the fact that ferredoxin 1 levels were found to be decreased in metronidazole-resistant E. histolytica [22]. At the moment, we do not have a conclusive explanation for these contradictory results, but it is conceivable that down-regulation of ferredoxin 1 in metronidazole-resistant E. histolytica could also be an accompanying effect of down-regulation of thioredoxin and thioredoxin reductase. As a potential parallel, thioredoxin has been shown to regulate a large number of proteins in plants, including enzymes involved in glycolysis such as aldolase, enolase, glyceraldehyde 3-phosphate dehydrogenase, and triose phosphate isomerase [40]. Thus, loss of thioredoxin activity or diminished thioredoxin levels in the cell could also impair the cellular metabolism, consequently leading to a down-regulation of ferredoxin.

Interestingly, a thioredoxin reductase originally with different annotation had been sequenced before the E. histolytica genome project had started. It was called disulphide oxidoreductase (Eh34) [41] or later, flavin reductase, [22], and was found to have reduced expression in metronidazole-resistant E. histolytica [22]. The very slight differences in the sequences of thioredoxin reductase from the genome project and Eh34 (2% on the DNA level) and the absence of an exact copy of the Eh34 gene in the genome database have not been resolved conclusively (I. Bruchhaus, personal communication). Eh34 was hypothesized by the authors to be involved in metronidazole activation because recombinant overexpression of Eh34 in E. histolytica rendered cells more vulnerable to metronidazole [22]. These unexpected findings of our colleagues strengthen our argument that thioredoxin reductase is involved in metronidazole activation in E. histolytica because down-regulation of thioredoxin reductase, as an enzyme known to be involved in oxidative stress response [42], would otherwise be highly counterproductive during metronidazole exposure that leads to the formation of reactive oxygen species, at least under microaerophilic conditions.

**Figure 6:**
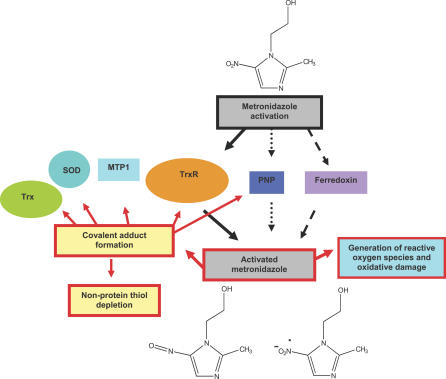
Model of Metronidazole Activation and Metronidazole Action in E. histolytica After uptake by the cell, metronidazole is reduced by thioredoxin reductase (TrxR) (as shown in this study; black arrow), possibly by purine nucleoside phosphorylase (hypothesized in this study; dotted arrow) and, probably, by ferredoxin (shown to reduce metronidazole in T. vaginalis and *G. intestinalis;* interrupted arrow). After activation, metronidazole can develop its toxicity in a 2-fold way, either as a nitroradical anion or, if further reduced, as a reactive nitrosoimidazole (red arrows). The nitroradical anion can reduce O_2_ and thereby generate reactive oxygen species, which are highly detrimental to the microaerophilic E. histolytica cell. Alternatively, the nitrosoimidazole is generated, which forms adducts with non-protein thiols and/or proteins, resulting in the depletion of non-protein thiols and the modification of thioredoxin reductase (TrxR), thioredoxin (Trx), superoxide dismutase (SOD), metronidazole target protein 1 (Mtp1), and purine nucleoside phosphorylase (PNP). Presumably, these five proteins become targets of modification due to their close spatial proximity to the site of metronidazole activation, i.e., thioredoxin reductase. The formation of covalent metronidazole adducts with proteins involved in antioxidant defense is likely to render the cells more vulnerable to oxidative stress, thereby exacerbating metronidazole toxicity to E. histolytica in the presence of oxygen [43].

The data gathered prompted us to propose a model of metronidazole action in E. histolytica that implies, apart from generation of reactive oxygen species in the presence of oxygen, that toxicity of metronidazole could be attributed to covalent adduct formation with essential thiols and the proteins described, leading to impaired protein function (Figure 6). Arguably, the formation of covalent adducts and oxidative stress could even intertwine, because metronidazole toxicity was shown to be exacerbated in E. histolytica under microaerophilic conditions as compared to metronidazole treatment in the complete absence of oxygen [43]. At first glance, this is counterintuitive, because our results show that higher oxygen levels lead to less non-protein thiol depletion (Figure 3A). Moreover, azomycin is by far less toxic to E. histolytica than metronidazole although it gives rise to more superoxide anion radicals (Table 2). However, azomycin forms fewer adducts with the five proteins identified, of which three, i.e., superoxide dismutase, thioredoxin reductase, and thioredoxin, are known to be involved in antioxidant defense. We show here that metronidazole-modified recEh TrxR displays a considerably reduced thioredoxin reductase activity. Thus, it is conceivable that the higher sensitivity of E. histolytica to metronidazole in the presence of oxygen is not due to a increased toxicity of metronidazole itself, but due to a reduced tolerance to oxygen because the cell's capability to remove harmful oxidants is impaired. Thioredoxin, for example, requires prior reduction by thioredoxin reductase in order to reduce peroxiredoxin [44,45], an enzyme that oxidizes hydrogen peroxide to water and oxygen. Since, in contrast to thioredoxin reductase activity, the nitroreductase activity of thioredoxin reductase is not diminished by metronidazole binding, superoxide radicals might be continuously generated after reduction of oxygen by activated nitroimidazoles. Superoxide dismutase breaks down superoxide radical anions to oxygen and hydrogen peroxide. The latter would then accumulate due to a reduced peroxiredoxin activity.

In any case, even in the complete absence of oxygen, metronidazole is a potent drug, suggesting that adduct formation of nitroimidazoles with the proteins identified and with non-protein thiol compounds can be very effective in killing E. histolytica cells (Figure 6). In addition, because mRNA expression was unchanged during metronidazole-exposure with 50 μM metronidazole (M. Tazreiter, unpublished data) and higher doses (e.g., 1 mM) of metronidazole rapidly led to the disintegration of cells, we do not believe that DNA damage plays a decisive role in short-term metronidazole-mediated toxicity.

Finally, because thioredoxin reductase is a ubiquitous enzyme, we suggest that our proposed model of metronidazole action might also, at least partly, apply for other organisms. Our preliminary data from 2D gels of, for example, T. vaginalis extracts corroborate the findings presented and discussed in this study, because thioredoxin reductase was identified among the proteins modified in this parasite.

## Materials and Methods

### Cell culture.

*E. histolytica* HM-1:IMSS cells were grown axenically at 36.5 °C in culture flasks that were completely filled with TYI-S-33 medium [46] and carefully sealed in order to ensure low oxygen tension. Culture medium was changed every 3 d. *Es. coli* BL21 (DE3) was grown in LB medium with appropriate antibiotics. Agar plates contained 15 g/l of agar.

When non-protein thiol levels were measured in E. histolytica while applying defined oxygen tensions, culture flasks were only half filled with growth medium, sealed with a vented plug, and then preincubated for 1 d either in tightly sealed jars containing Merck Anaerocult A (Merck, http://www.merck.de/) for anaerobic conditions (0% O_2_ and 18% CO_2_) or Merck Anaerocult C for microaerophilic conditions (5% O_2_ and 8% CO_2_) or in a normal incubator in the presence of air (21% O_2_ and 0.4% CO_2_) for aerobic conditions. Cell cultures were then harvested, followed by the resuspension of the cell pellets in the respective preincubated media and the exposure to metronidazole or to azomycin as stated below.

### Cell harvest and preparation of cell lysates.

Cells were harvested by centrifugation at 500*g* at room temperature for 5 min and then washed twice with PBS to remove residual serum components. Cell lysates for 2DE were prepared as described previously [39]. Briefly, proteins were precipitated with trichloroacetic acid and acetone, and solubilized in a classical buffer containing urea, thiourea, CHAPS, and DTT.

### 2DE and image analysis.

2DE was performed as described previously [39]. Analytical gels were silver stained [47], whereas preparative gels were stained with Coomassie Brilliant Blue R-250. After staining, gels were scanned with an Epson 1680 Pro scanner (Epson, http://www.epson.com/) and analyzed with the Melanie 2D gel analysis software (GeneBio, http://www.genebio.com/).

### Protein identification and mass spectrometry of intact proteins.

The excised 2DE spots were destained, digested with trypsin, and analyzed by LC-ESI-QTOF-MS as described previously [48]. For protein identification, the MS/MS data were subjected to database search against the SwissProt database using the Mascot search engine (http://www.matrixscience.com/) and Protein Global Server 2.1 (Waters-Micromass, http://www.waters.com/). A compilation of mass data is given in Figures S1–S5.

The mass of intact proteins with and without metronidazole or tinidazole treatment (see above) was determined using LC-ESI-QTOF-MS. An aliquot corresponding to approx 500–1,000 pmol of purified protein (recombinantly produced in *Es. coli*) was subjected to liquid chromatography (LC) using a BioBasic C4 column (30 × 0.32 mm; Thermo Electron Corporation, http://www.thermo.com/) using a CAP-LC (Waters-Micromass). Proteins were loaded onto the column in solvent A (water containing 0.1% formic acid) and eluted using a gradient from 0%–70% solvent B (acetonitrile containing 0.1% formic acid) in 30 min. The flow rate was held at 5 μl/min throughout the analysis. Instrument calibration and tuning in the mass range from 500–4,000 Da was achieved using a 2 mg/ml solution of sodium iodide in 50% isopropanol. Spectra were deconvoluted using MaxEnt1 function of the MassLynx 4.0 SP4 software (Waters-Micromass).

### Estimation of non-protein thiol concentrations in metronidazole-treated and untreated cells.

The levels of non-protein thiols in metronidazole-treated, azomycin-treated, or in untreated cells were determined as described [49]. Briefly, cells were harvested and washed in 20 mM EDTA. Pellets were resuspended in 20 mM EDTA, and cells were disrupted by repeated freezing and thawing. Cell debris was removed by centrifugation at 20,000*g* for 15 min. After measuring protein levels with Bradford reagent, equal amounts of protein were precipitated in 5% TCA at room temperature, followed by centrifugation at 20,000*g* for 15 min. Supernatants containing the non-protein thiols were removed, and the double volume of 0.4 M Tris/HCl (pH 8.9) was added. A total of 1.7 μl 100 mM 5,5′-dithiobis-(2-nitrobenzoic acid) (DTNB) per ml reaction mixture was added. Reduction of DTNB was measured at λ = 412 nm in a Jenway 6505 UV/Vis spectrometer (Δɛ_412_ = 13.6 mM^−1^ cm^−1^; http://www.jenway.com/en/index.php).

### Cysteine protection assay.

Before addition of various amounts of metronidazole and/or cysteine, total and viable cell numbers were determined by trypan blue exclusion in a Bürker-Türk hemocytometer. After addition of reagents, cells were incubated for 20 h at 36.5 °C. After incubation, total and viable cell numbers were redetermined.

### Expression of recombinant hexahistidine-tagged thioredoxin and thioredoxin reductase in *Es. coli*.

The genes for thioredoxin reductase and thioredoxin were amplified from genomic E. histolytica HM-1:IMSS DNA. Primers were 5′-TAC GTA CGC ATA TGA GTA ATA TTC ATG ATG TTG TGA TTA TCG GC-3′ (TrxR forward) and 5′-TCA TCC AGC TCG AGT TAG TGG TGA TGG TGA TGA TGA GTT TGA AGC CAT TTT TCA CAG-3′ (TrxR reverse) for thioredoxin reductase, and 5′-TAC GTA CGC ATA TGG CTG TAC TTC ATA TTA ACG CTC TTG ATC AA-3′ (Trx forward) and 5′-TCA TCC AGC TCG AGT TAG TGA TGG TGA TGG TGA TGT CGT GTT TCA ACC ATT TGT TTT AAG GCA-3′ (Trx reverse) for thioredoxin. Forward primers include an NdeI restriction site, whereas reverse primers bear an XhoI restriction site and a hexahistidine tag for convenient protein isolation. PCR fragments were ligated into the pET 17b vector (Novagen/VWR, http://www.emdbiosciences.com/html/NVG/home.html). The plasmid sequences were confirmed on both strands by using T7 and pET reverse primers (GATC Biotech, http://www.gatc-biotech.com/en/index.php). The confirmed plasmids were transfected into *Es. coli* BL21(DE3) cells. Transformants were selected on 20 μg/ml ampicillin. Expression of recombinant proteins was induced by addition of 0.5 mM IPTG. If proteins were to be modified with metronidazole, 1 mM metronidazole was added to the LB medium, and cells were grown in completely filled tissue culture flasks under exclusion of air. Three hours after induction, cells were harvested and then disrupted by vigorous grinding in a mortar. Subsequently, recombinant proteins were purified via Ni-NTA spin columns (Qiagen, http://www1.qiagen.com/). Recombinantly expressed E. histolytica thioredoxin and E. histolytica thioredoxin reductase are referred to as recEh Trx, and recEh TrxR, respectively.

### Assaying disulfide reductase activity of recEh TrxR.

The assay was performed as described elsewhere [50] using recEh Trx and recEh TrxR in combination. The reaction buffer contained 100 mM potassium phosphate (pH 7.5), 1 mM EDTA, 1 mM DTNB, and 0.5 mM NADPH. All reactions were done with 2 μg/ml recEh Trx and 5 μg/ml recEh TrxR. RecEh TrxR oxidized NADPH to NADP in order to reduce the disulfide bond at the active site of recEh Trx, which, in turn, reduced DTNB. Ongoing reduction of DTNB was measured at λ = 412 nm (Δɛ_412_ = 13.6 mM^−1^ cm^−1^) over a period of 5 min at 25 °C.

### Assaying nitroreductase activity of recEh TrxR.

Reduction of CDNB by Eh TrxR was measured by determining NADPH consumption at λ = 340 nm (Δɛ_340_ = 6.2 mM^−1^ cm^−1^). The reaction buffer contained 100 mM potassium phosphate (pH 7.5), 1 mM EDTA, and 0.1 mM NADPH. Reduction of CDNB was determined at a concentration of 100 μM. For reasons of practicability, because nitroimidazoles display high absorbances at λ = 340 nm and thereby heavily disturb the assay, reduction of nitroimidazoles was measured in a modified assay [30] via reduction of cytochrome c at λ = 550 nm (Δɛ_550_ = 20 mM^−1^ cm^−1^), either directly by reduced nitro compounds (CDNB, azomycin, and metronidazole), or indirectly by superoxide radical anions that are generated when nitroradicals transfer an electron to molecular oxygen in the reaction buffer. In any case, nitro compounds had been previously reduced by recEh TrxR. Therefore, it was assumed that reduction of one nitro group by Eh TrxR subsequently resulted in one reduced cytochrome c molecule. Reaction mixtures contained 100 mM potassium phosphate (pH 7.5), 1 mM EDTA, 0.5 mM NADPH, 50 μM cytochrome c, 4 μg/ml recEh TrxR (equal to 118 nM) and different amounts of CDNB, azomycin, or metronidazole, respectively. Reduction of cytochrome c was measured over a time span of 5 min at 25 °C. In order to assess the proportion of electrons that are directly transferred from the reduced nitro compounds to cytochrome c, 2.5 μM bovine erythrocyte superoxide dismutase (i.e., an approximately 20-fold excess over Eh TrxR) were added to the reactions to remove all generated superoxide radical anions. Bovine erythrocyte superoxide dismutase was purchased from Sigma (http://www.sigmaaldrich.com/).

## Supporting Information

Figure S1 Sequence of E. histolytica Superoxide Dismutase (P34107) and Peptides Identified by MS/MS (A) Based on the MS/MS spectrum and the particular mass differences, the Ser in position two was identified to be acetylated after removal of the amino-terminal Met. The identified peptide sequences are in red.
(B) MS/MS spectrum of the tryptic peptide 2–21. Ser in position 2 was amino-terminally acetylated after removal of the Met.
(C) MS/MS spectrum of peptide 97–109.
(D) MS/MS spectrum of peptide 171–184.
(45 KB PDF)

Figure S2 Sequence of E. histolytica Metronidazole Target Protein 1 (Q50UL1) and Peptides Identified by MS/MS (A) MS/MS spectrum of peptide 43–57. The identified peptide sequences are in red.
(B) MS/MS spectrum of peptide 58–77.
(38 KB PDF)

Figure S3 Sequence of E. histolytica Thioredoxin Reductase (Q50PB3) and Peptides Identified by MS/MS (A) Based on the MS/MS spectrum and the particular mass differences, the Ser in position 2 was identified to be acetylated after removal of the amino-terminal Met. The identified peptide sequences are in red.
(B) MS/MS spectrum of peptide 2–25. Ser in position 2 was amino-terminally acetylated after removal of the Met.
(C) MS/MS spectrum of peptide 261–273.
(D) MS/MS spectrum of the carboxy-terminal peptide 292–314.
(50 KB PDF)

Figure S4 Sequence of E. histolytica Thioredoxin (Q51ER4) and Peptides Identified by MS/MS (A) Based on the MS/MS spectrum and the particular mass differences, the Ala in position 2 was identified to be acetylated after removal of the amino-terminal Met. The identified peptide sequences are in red.
(B) MS/MS spectrum of peptide 2–20. Ala in position 2 was amino-terminally acetylated after removal of the Met.
(C) MS/MS spectrum of peptide 36–52.
(35 KB PDF)

Figure S5 Sequence of E. histolytica Purine Nucleoside Phosphorylase (Q51AY9) and Peptides Identified by MS/MS (A) On the amino terminus, the first two amino acids (Met and Cys) appear to be removed because a peptide corresponding to positions 3–13 was identified. After removal of Met1 and Cys2, amino-terminal Ala at position 3 is apparently acetylated, as already observed in most of the other samples. The identified peptide sequences are in red.
(B) MS/MS spectrum of peptide 3–13. Ala in position 3 was amino-terminally acetylated after removal of the Met and Cys.
(C) MS/MS spectrum of peptide 61–75.
(D) MS/MS spectrum of peptide 288–311.
(50 KB PDF)

### Accession Numbers

The National Center for Biotechnology Information (http://www.ncbi.nlm.nih.gov/) accession numbers for the proteins discussed in this paper are as follows: α-amylase (XP_652601), metronidazole target protein 1 (XP_650662), purine nucleoside phosphorylase (XP_655398), superoxide dismutase (XP_648827), thioredoxin (XP_656726), and thioredoxin reductase (XP_655748).

## Abbreviations

2D: two-dimensional
2DE: two-dimensional gel electrophoresis
LC-ESI-QTOF-MS: liquid chromatography electrospray ionization quadrupole time-of-flight tandem mass spectrometry
MS/MS: tandem mass spectrometry
PFOR: pyruvate:ferredoxin oxidoreductase
pI: isoelectric point
recEh Trx: recombinant *Entamoeba histolytica* thioredoxin
recEh TrxR: recombinant *Entamoeba histolytica* thioredoxin reductase
