# Supplementary material for: Nitroimidazole Action in Entamoeba histolytica: A Central Role for Thioredoxin Reductase
Source: PLoS Biol. 2007 Jul 31;5(8):e211. doi: 10.1371/journal.pbio.0050211 (PMC1933457; doi:10.1371/journal.pbio.0050211)
Supplement: Supplementary file 1 [file pbio.0050211.sg001.pdf]

A

SwissProt Entry P34107, Superoxide dismutase

10 20 30 40 50 60

M**SFQLPQLPY AYNALEPHIS K**ETLEFHHDK HHATYVNKLN GLVKGTEQEH K**TLEELIKQK**

70 80 90 100 110 120

PTQAIYNNAA QAWNHAFYWK CMCGCGVKPS EQLIAK**LTAA FGGLEEFKK**K FTEK**AVGHFG**

130 140 150 160 170 180

SGWCWLVEHD GKLEIIDTHD AVNPMTNGMK PLLTCDVWEH AYYIDTRNNR **AAYLEHWWNV**

190

VNWKFVEEQL

B

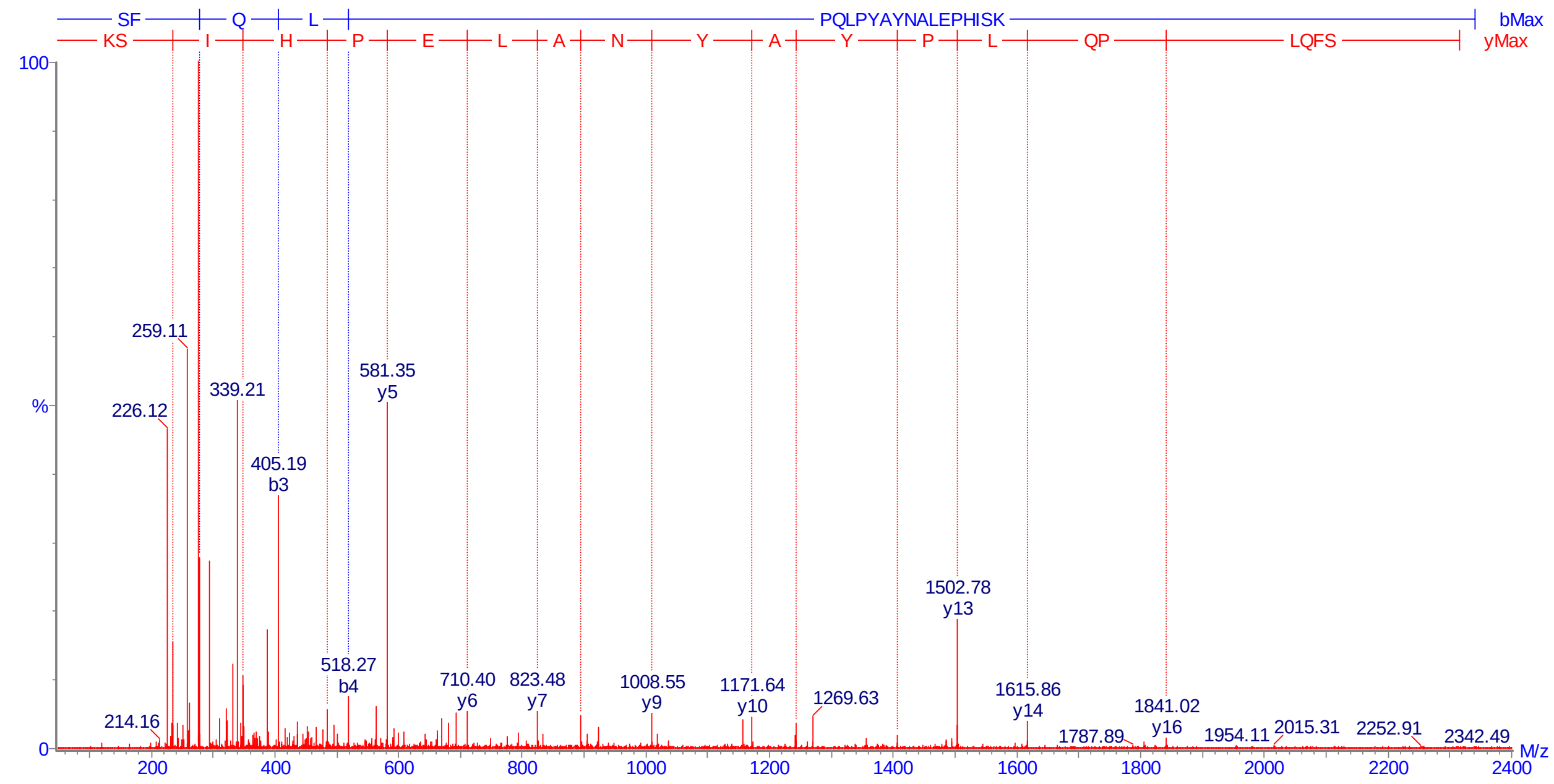

C

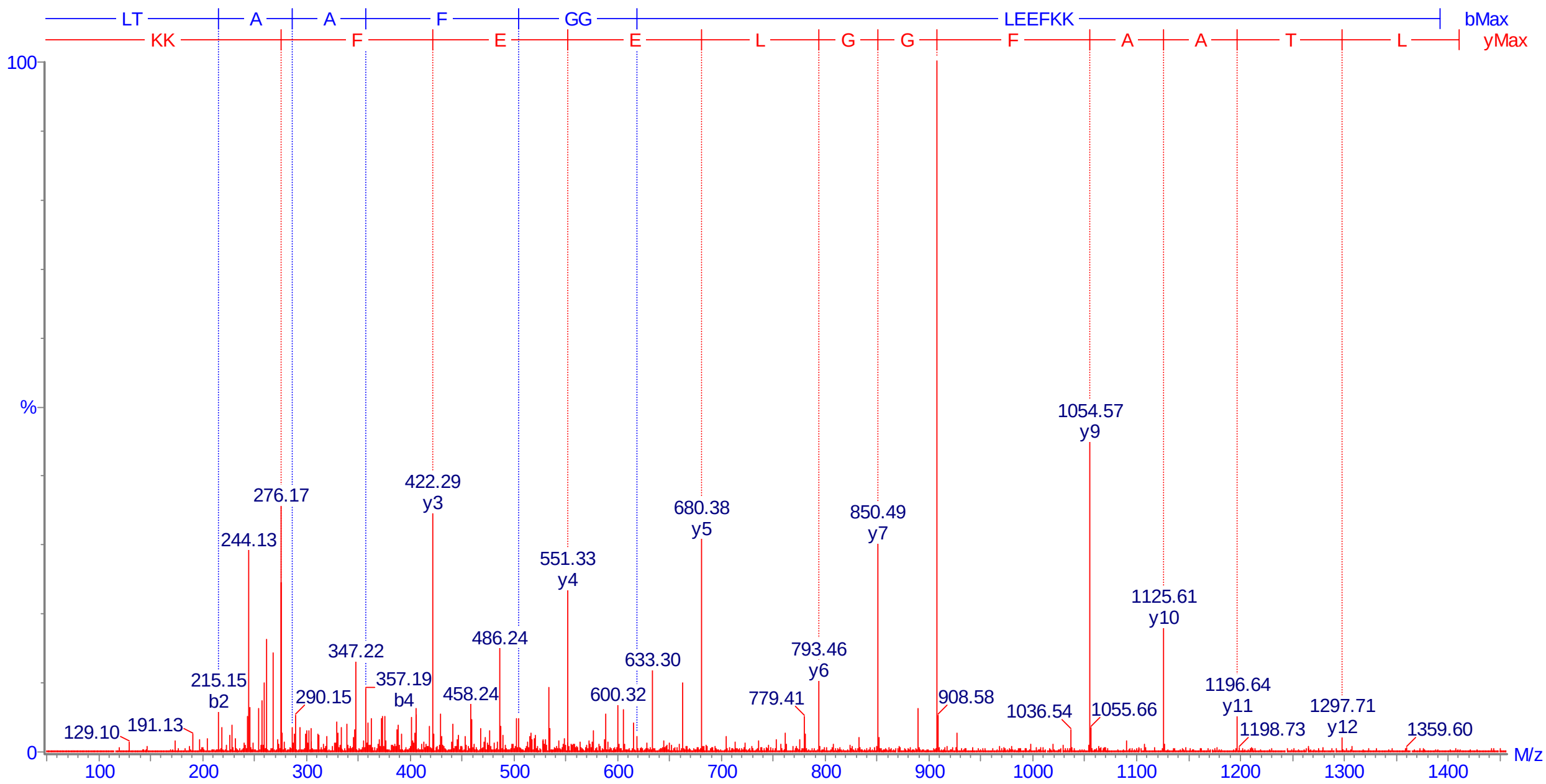

LT
A
A
F
GG
LEEFKK
bMax
KK
F
E
E
L
G
G
F
A
A
T
L
yMax
100
%
0
1054.57
y9
422.29
y3
276.17
680.38
y5
850.49
y7
244.13
551.33
y4
1125.61
y10
486.24
347.22
633.30
793.46
y6
215.15
b2
357.19
b4
290.15
458.24
600.32
779.41
908.58
1196.64
y11
1036.54
1055.66
1297.71
y12
129.10
191.13
1198.73
1359.60
M/z

D

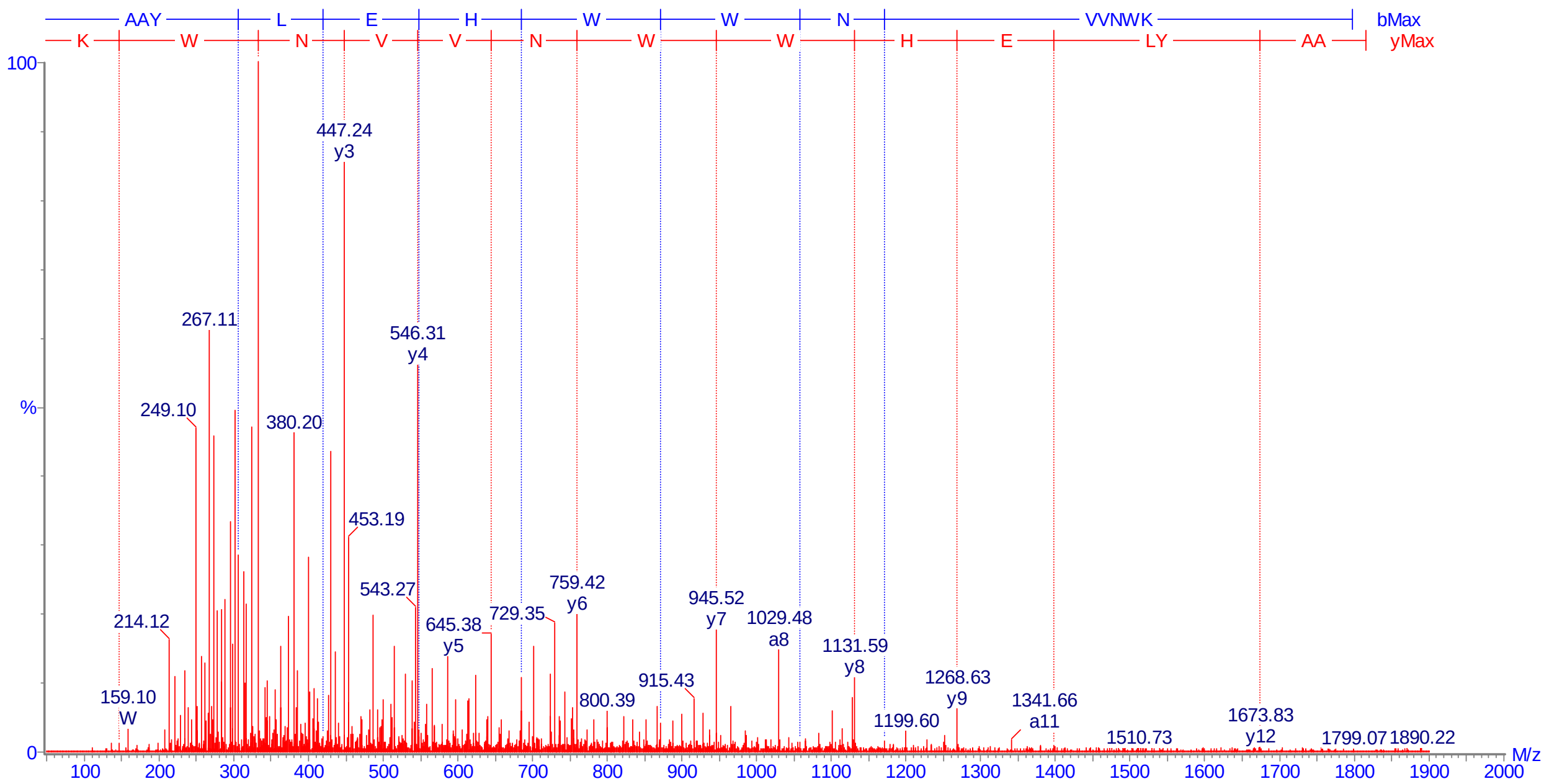

AAY
L
E
H
W
W
N
VVNWK
bMax
K
W
W
N
V
V
N
W
W
H
E
LY
AA
yMax
100
%
0
447.24
y3
267.11
546.31
y4
249.10
380.20
453.19
543.27
759.42
y6
214.12
645.38
y5
729.35
945.52
y7
1029.48
a8
1131.59
y8
915.43
1268.63
y9
159.10
W
800.39
1199.60
1341.66
a11
1673.83
y12
1510.73
1799.07
1890.22
M/z
