# Supplementary material for: Nitroimidazole Action in Entamoeba histolytica: A Central Role for Thioredoxin Reductase
Source: PLoS Biol. 2007 Jul 31;5(8):e211. doi: 10.1371/journal.pbio.0050211 (PMC1933457; doi:10.1371/journal.pbio.0050211)
Supplement: Supplementary file 2 [file pbio.0050211.sg002.pdf]

A

SwissProt Entry Q50UL1,
Hypothetical protein, 205.t00008 "metronidazole target protein 1"

```
        10         20         30         40         50         60
```
MVHIIFIIDY K**THPGQVVR**V CGSAK**ELGSW** **TEGYTMTYK**D GK**CIAEVDIN** **TIPFEYKFQV**

```
        70         80         90        100        110        120
```
YNCDGHYVEQ **WESCANR**LFI LCK**QADEIVV** **ESVWNYPDGT** **K**ISSKRTKIV KSTIKKSCSQ

```
  125
```
EFADL

B

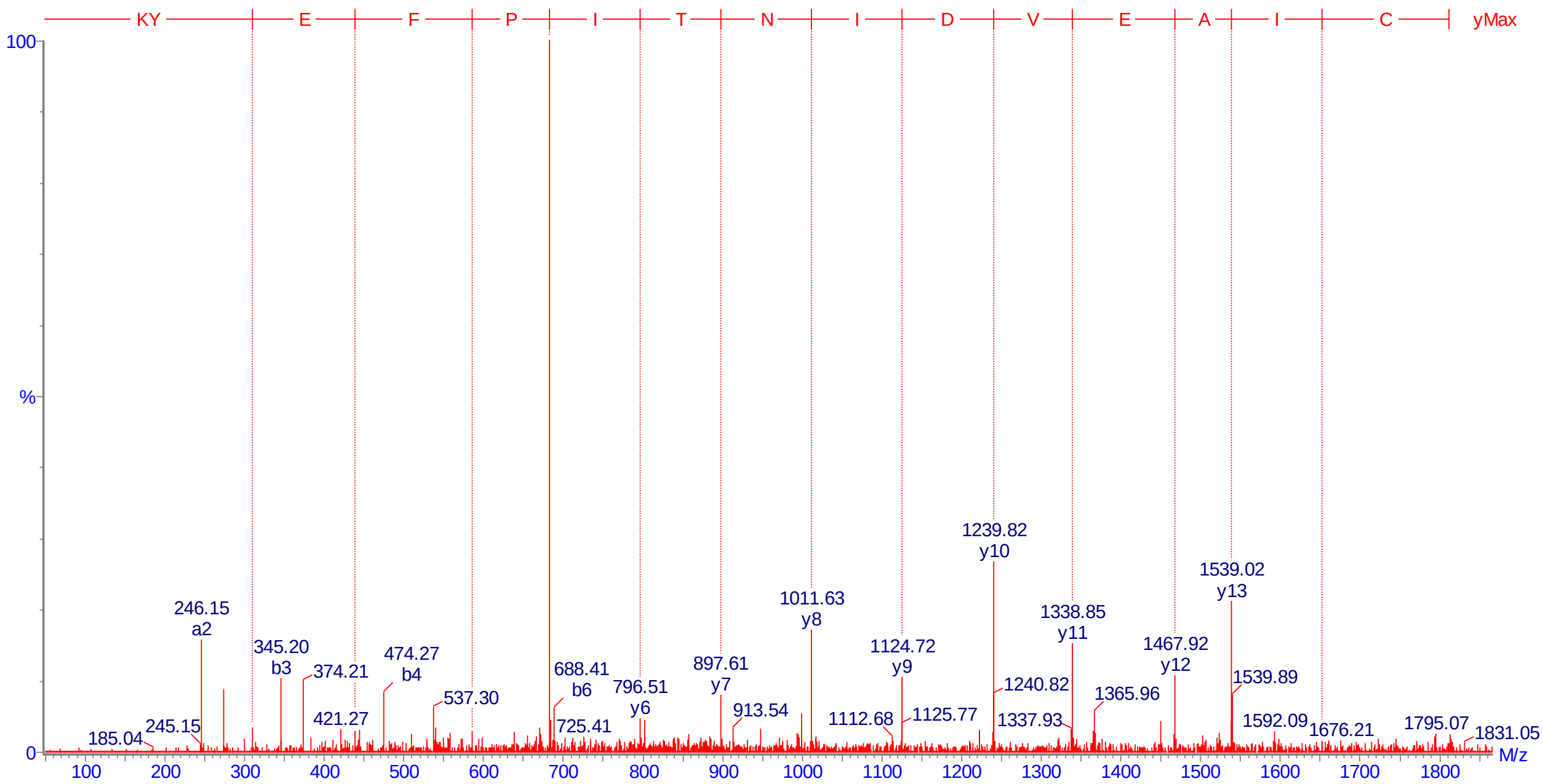

C

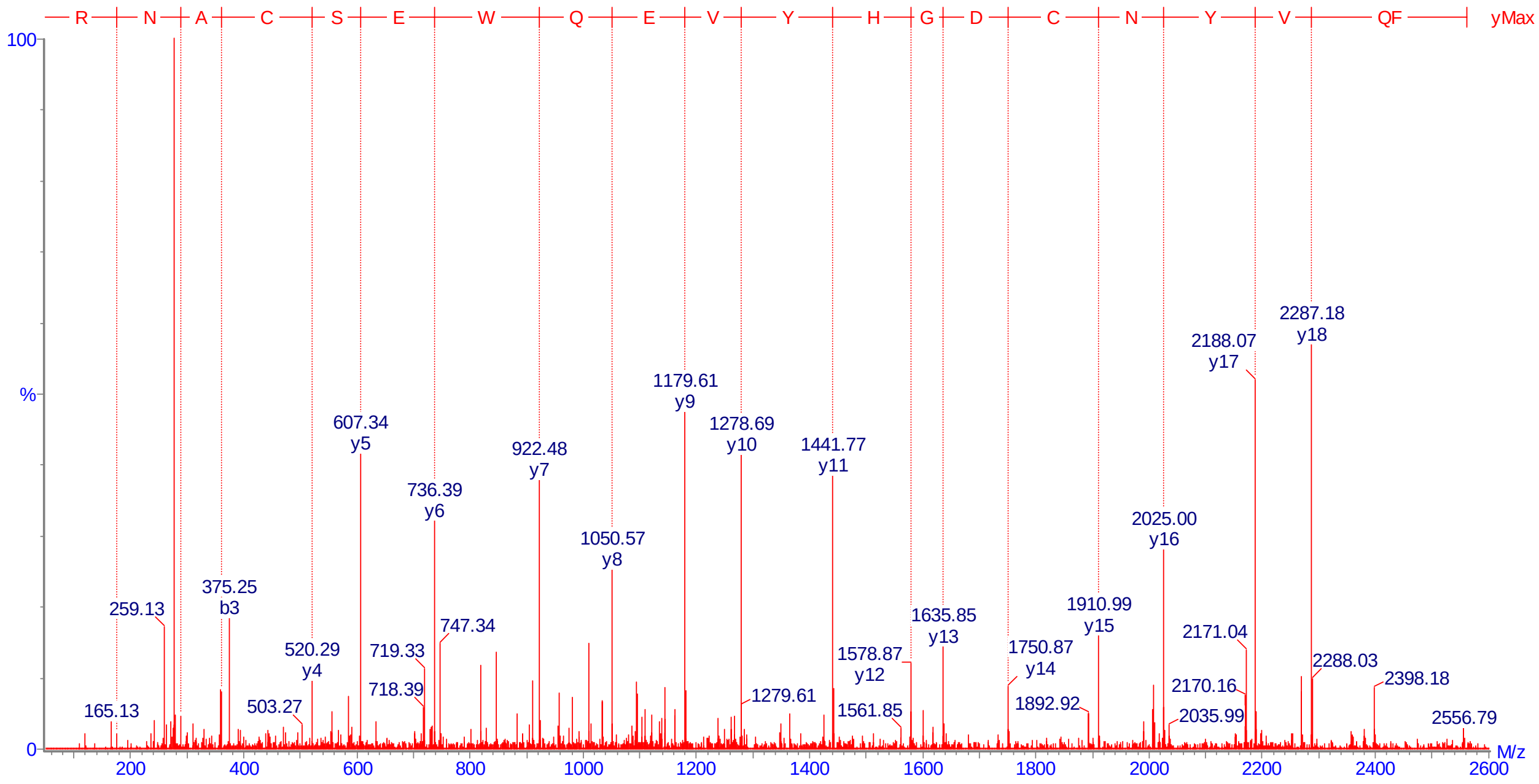

R N A C S E W Q E V Y H G D C N Y V QF
yMax
100
%
0
259.13
165.13
375.25
b3
520.29
y4
503.27
607.34
y5
719.33
718.39
736.39
y6
747.34
922.48
y7
1050.57
y8
1179.61
y9
1278.69
y10
1279.61
1441.77
y11
1578.87
y12
1561.85
1635.85
y13
1750.87
y14
1892.92
1910.99
y15
2025.00
y16
2035.99
2170.16
2171.04
2188.07
y17
2287.18
y18
2288.03
2398.18
2556.79
200
400
600
800
1000
1200
1400
1600
1800
2000
2200
2400
2600
M/z
