# Supplementary material for: Nitroimidazole Action in Entamoeba histolytica: A Central Role for Thioredoxin Reductase
Source: PLoS Biol. 2007 Jul 31;5(8):e211. doi: 10.1371/journal.pbio.0050211 (PMC1933457; doi:10.1371/journal.pbio.0050211)
Supplement: Supplementary file 3 [file pbio.0050211.sg003.pdf]

A

SwissProt Entry Q50PB3, Thioredoxin reductase

10 20 30 40 50 60

M**SNIHDVVII GSGPAAHTAA IYLGR**SSLKP VMYEGFMAGG VAAGGQLTTT TIIENFPGFP

70 80 90 100 110 120

NGIDGNELMM NMRTQSEK**YG TTIITETIDH VDFSTQPFKL FTEEGKEVLT K**SVIIATGAT

130 140 150 160 170 180

AKR**MHVPGED KYWQNGVSAC AICDGAVPIF R**NK**VLMVVGG GDAAMEEALH LTK**YGSKVII

190 200 210 220 230 240

LHRRDAFRAS KTMQERVLNH PK**IEVIWNSE LVELEGDGDL LNGAK**IHNLV SGEYK**VVPVA**

250 260 270 280 290 300

GLFYAIGHSP NSKFLGGQVK **TADDGYILTE GPKTSVDGVF ACGDVCDR**VY R**QAIVAAGSG**

310

CMAALSCEKW LQTH

B

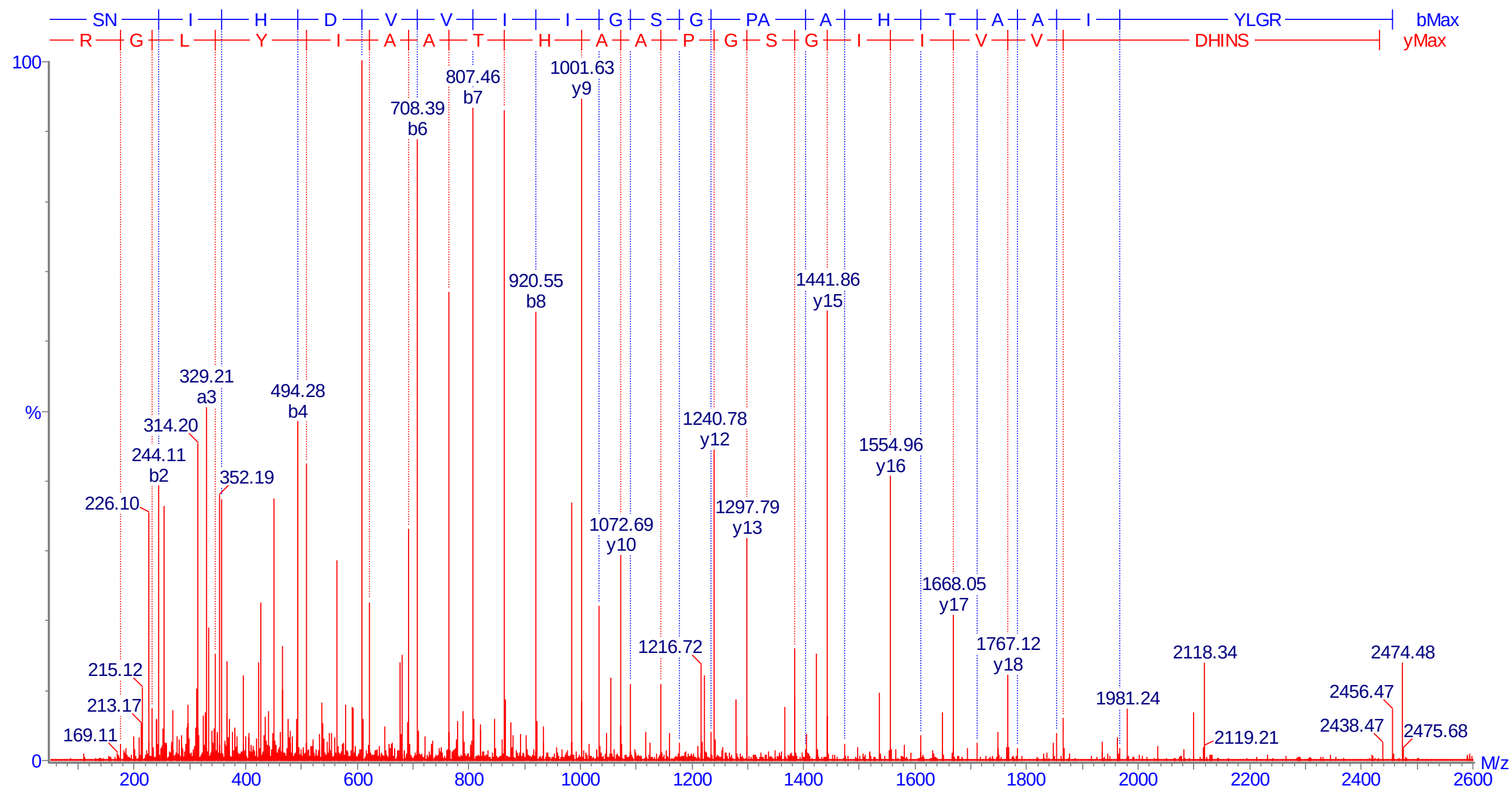

C

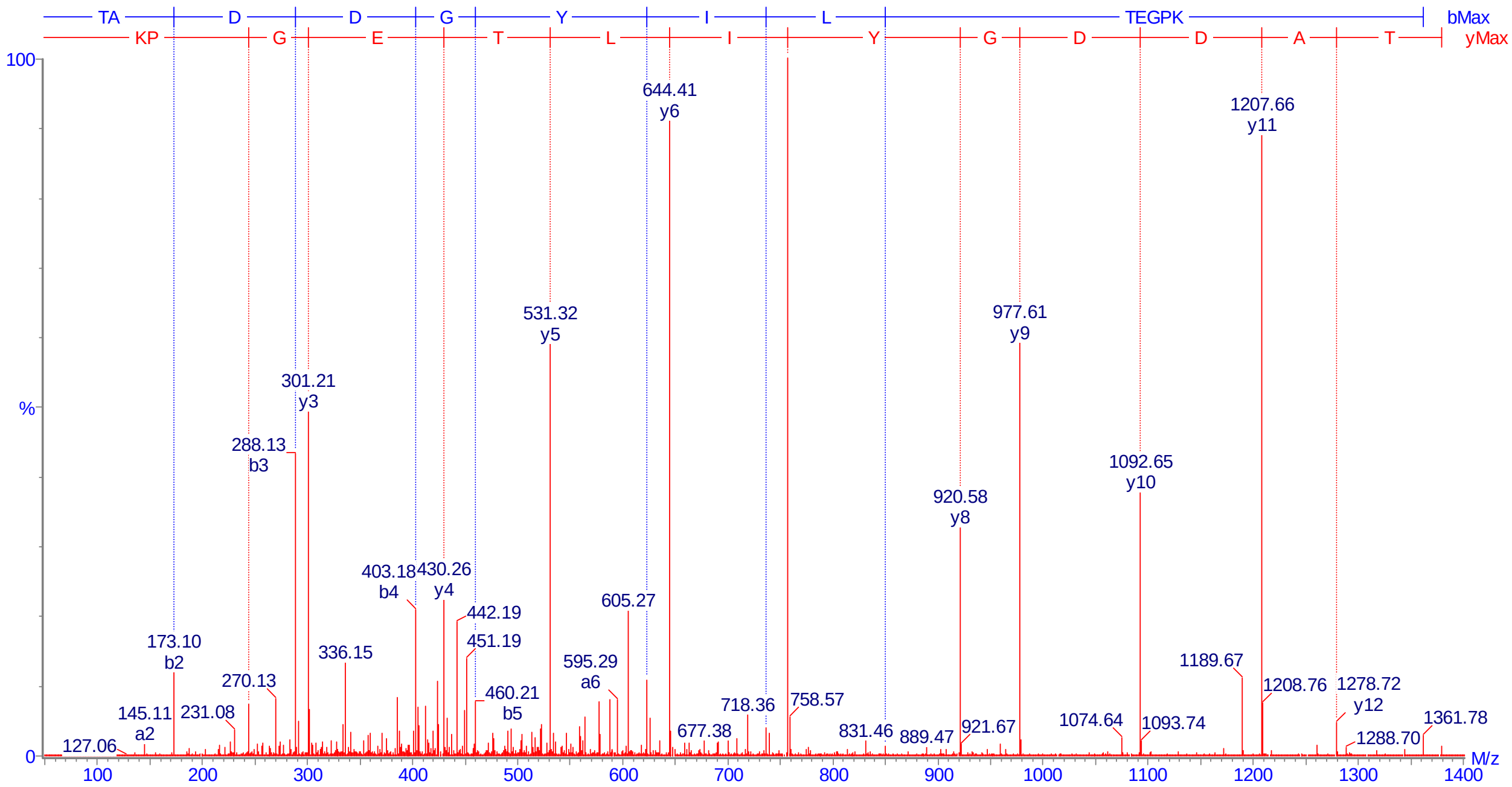

TA
D
D
G
Y
I
L
TEGPK
bMax
KP
G
E
T
L
I
Y
G
D
D
A
T
yMax
100
%
0
644.41
y6
1207.66
y11
531.32
y5
977.61
y9
301.21
y3
288.13
b3
1092.65
y10
920.58
y8
403.18
b4
430.26
y4
442.19
451.19
605.27
173.10
b2
336.15
595.29
a6
1189.67
270.13
460.21
b5
1208.76
1278.72
y12
145.11
a2
231.08
718.36
758.57
1361.78
127.06
677.38
831.46
889.47
921.67
1074.64
1093.74
1288.70
M/z

D

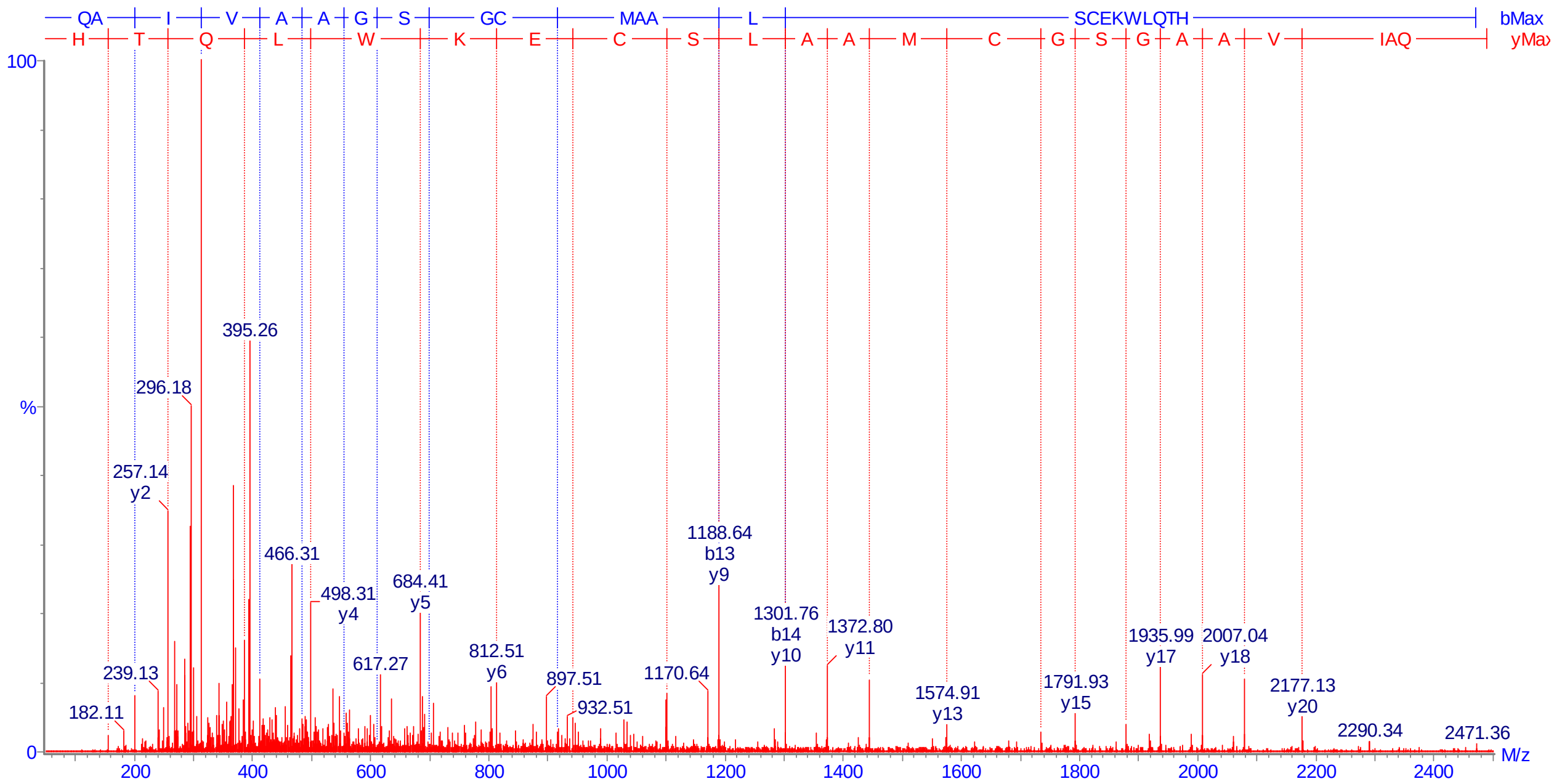

QA
I
V
A
A
G
S
GC
MAA
L
SCEKWLQTH
bMax
H
T
Q
L
W
K
E
C
S
L
A
A
M
C
G
S
G
A
A
V
IAQ
yMax
100
%
0
395.26
296.18
257.14
y2
1188.64
b13
y9
466.31
498.31
y4
684.41
y5
1301.76
b14
y10
1372.80
y11
1935.99
y17
2007.04
y18
239.13
617.27
812.51
y6
897.51
1170.64
1574.91
y13
1791.93
y15
2177.13
y20
182.11
932.51
2290.34
2471.36
M/z
