# Supplementary material for: Nitroimidazole Action in Entamoeba histolytica: A Central Role for Thioredoxin Reductase
Source: PLoS Biol. 2007 Jul 31;5(8):e211. doi: 10.1371/journal.pbio.0050211 (PMC1933457; doi:10.1371/journal.pbio.0050211)

A

SwissProt Entry Q51ER4, Thioredoxin

```
         10         20         30         40         50         60
MAVLHINALD QLTALLSTEK VIVIDFFATW CGPCRSISPY FEELAGQYNN IKFVKVDVDQ

         70         80         90        100
AEEICVNYKV RSMPTFVLVK DGIEQKRFSG ADRNALKQMV ETA
```

B

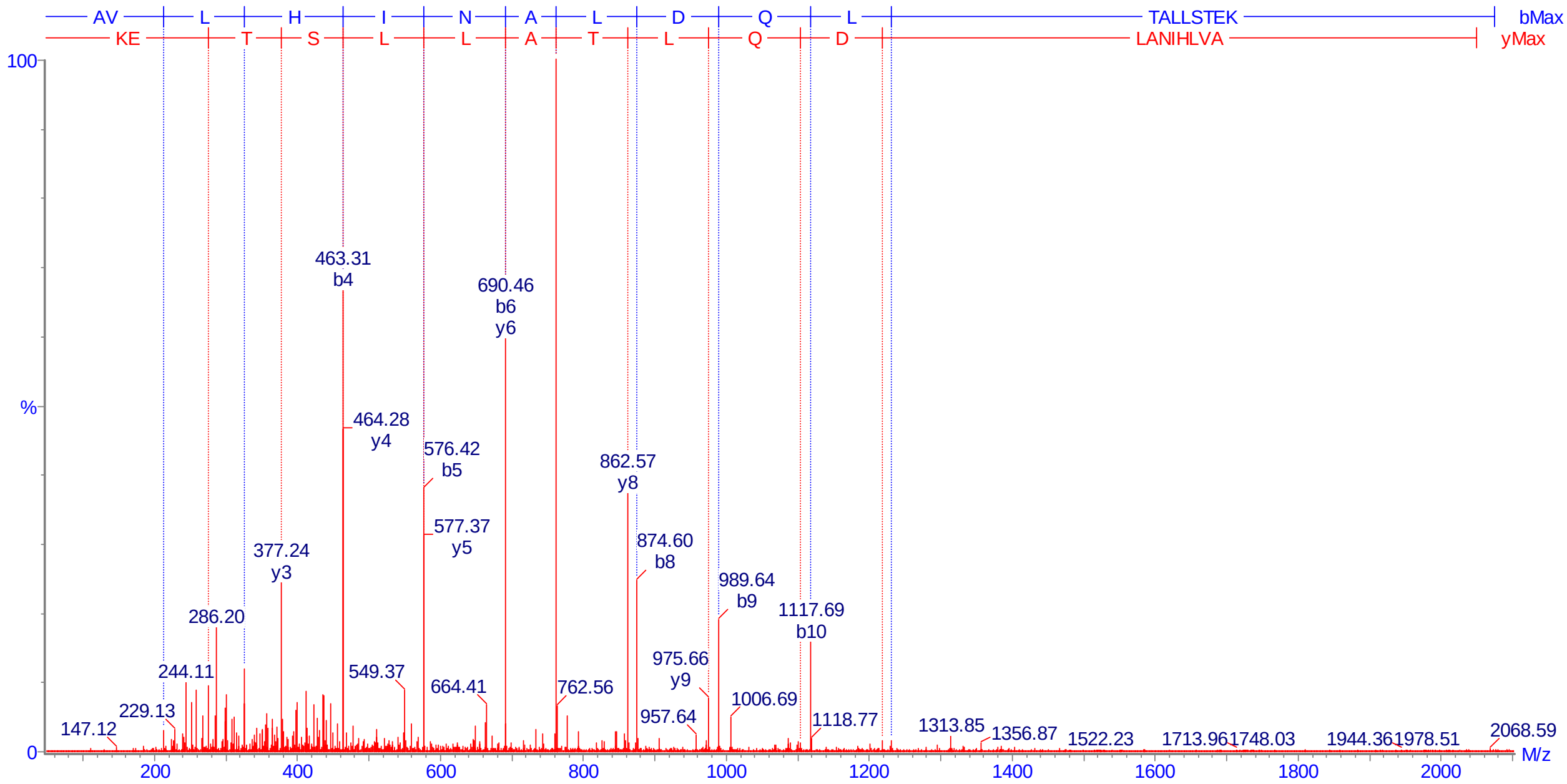

C

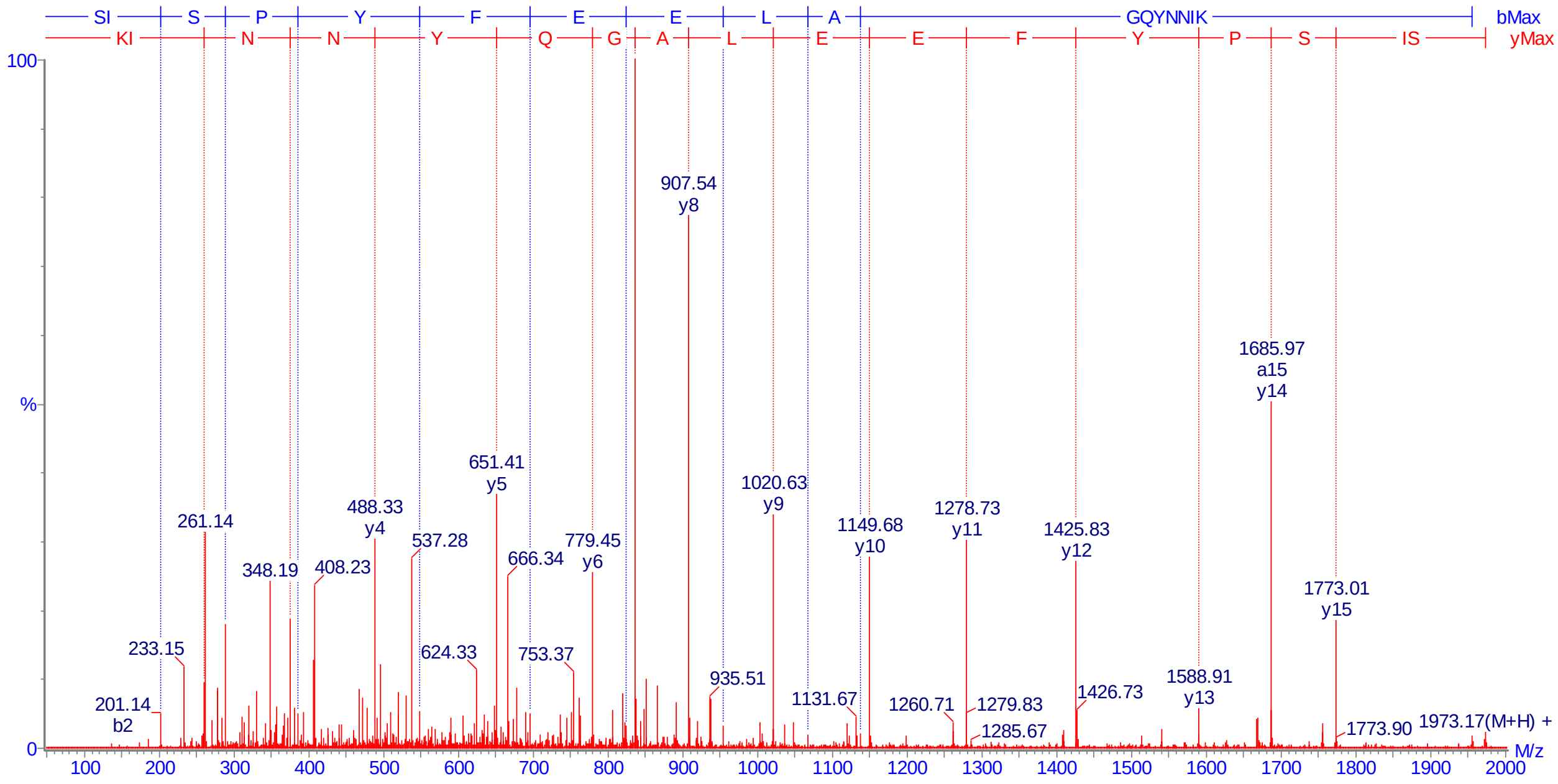

Supplement: Supplementary file 4 [file pbio.0050211.sg004.pdf]
