# Supplementary material for: Nitroimidazole Action in Entamoeba histolytica: A Central Role for Thioredoxin Reductase
Source: PLoS Biol. 2007 Jul 31;5(8):e211. doi: 10.1371/journal.pbio.0050211 (PMC1933457; doi:10.1371/journal.pbio.0050211)
Supplement: Supplementary file 5 [file pbio.0050211.sg005.pdf]

A

SwissProt Entry Q51AY9, Purine nucleoside phosphorylase

```
        10         20         30         40         50         60
MCANINEATL PAKNGVIYHL QVTANDVANN IIMVGDPERV PKIAELVFDK SKDIFRKDHR

        70         80         90        100        110        120
GLCVMTGYTI GDGMRLSVIT HGMGTGSAEI VLNEVLALKA IDINSRTPIE VKEPVNIIRI

       130        140        150        160        170        180
GTSGALQSST KLGTTIITKY SIGLDNTGLF YDIPPQSPQI VKLENIVDDL FNKGAANTCR

       190        200        210        220        230        240
YKGGIHAYAS QPDPRMVDAL VRASEKNKLL HKLGITTTVP GFFNCQGRLL FEELPETVES

       250        260        270        280        290        300
IDKLLCFDFD GVQPENLEME ISILSLISSG LKWVRFGAMC MAIANRRLNT FASDVESSLD

       310      318
PTVLTAVDAL REMSKIPL
```

Highlighted (red, bold) residues: MC**ANINEATL** **PAK**NGVIYHL QVTANDVANN IIMVGDPERV PK**IAELVFDK** SKDIFRKDHR **GLCVMTGYTI** **GDGMRLSVIT** **HGMGTGSAEI** **VLNEVLALKA** **IDINSRTPIE** **VKEPVNIIRI** **GTSGALQSST** **KLGTTIITKY** **SIGLDNTGLF** **YDIPPQSPQI** **VK**LENIVDDL FNKGAANTCR YKGGIHAYAS QPDPRMVDAL VRASEKNKLL HK**LGITTTVP** **GFFNCQGRLL** **FEELPETVES** **IDK**LLCFDFD GVQPENLEME ISILSLISSG LKWVR**FGAMC** **MAIANRRLNT** **FASDVESSLD** **PTVLTAVDAL** **R**EMSKIPL

B

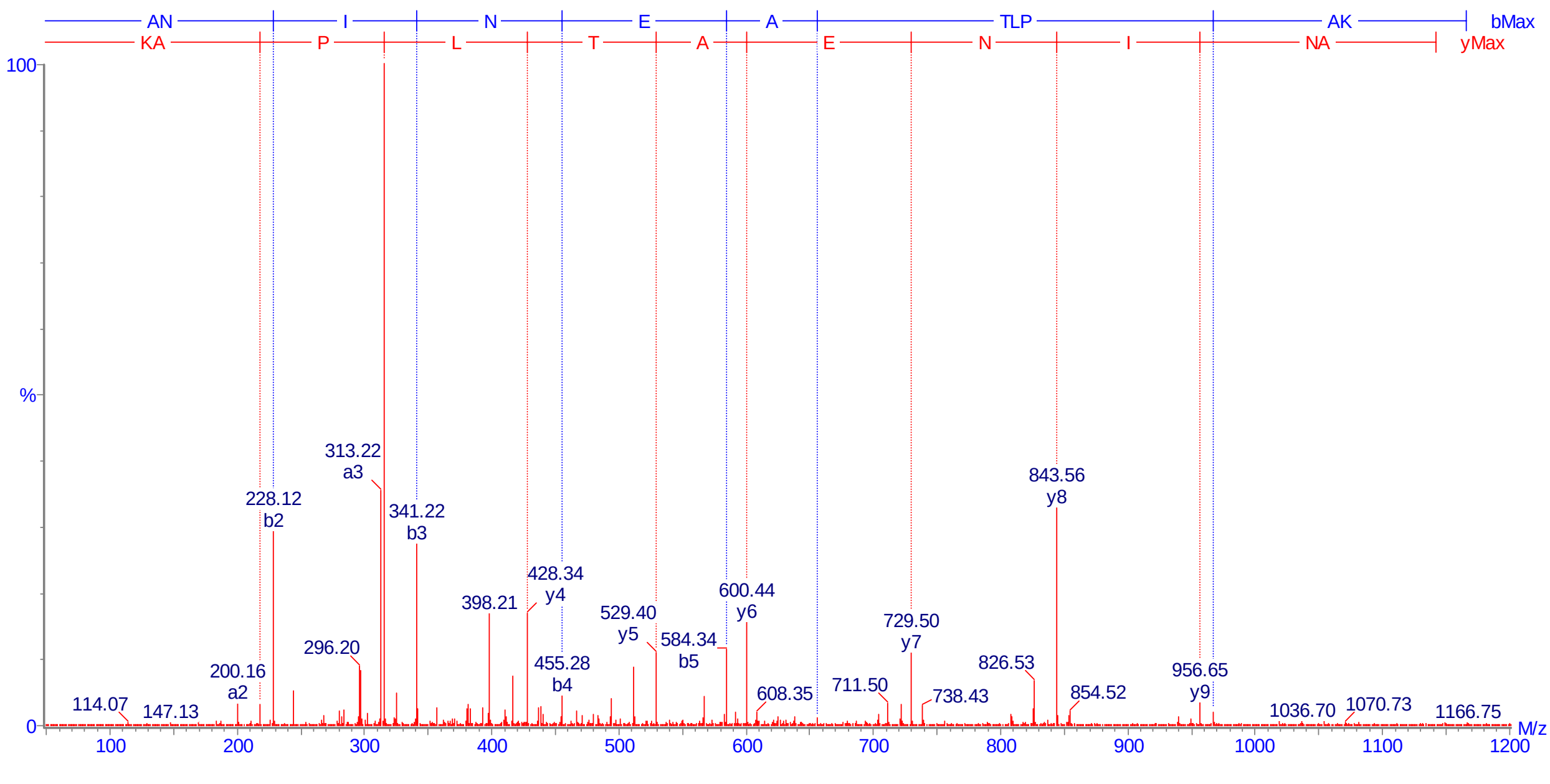

C

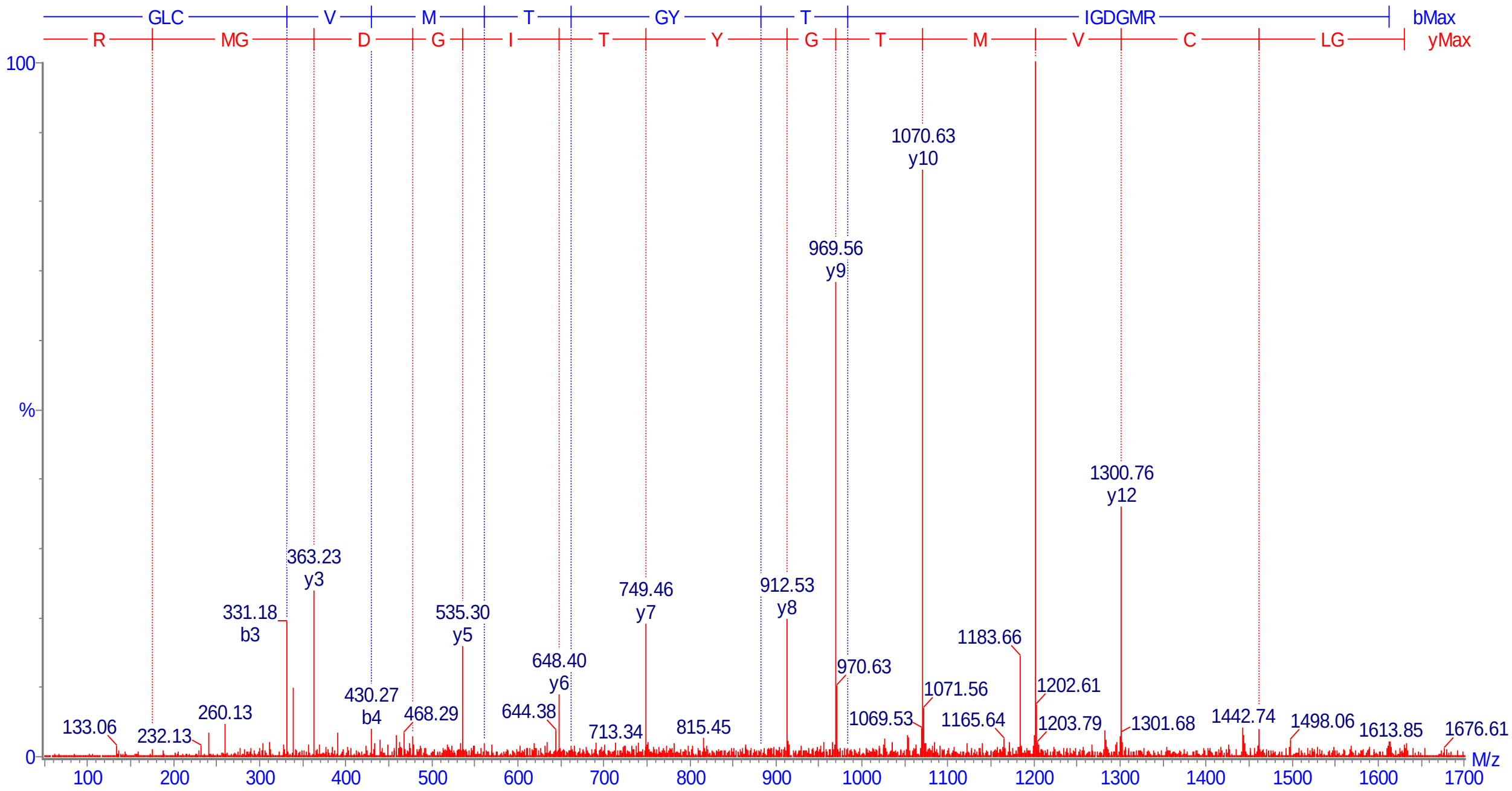
GLC V M T GY T IGDGMR bMax
R MG D G I T Y G T M V C LG yMax
100
%
0
133.06
232.13
260.13
331.18 b3
363.23 y3
430.27 b4
468.29
535.30 y5
644.38
648.40 y6
713.34
749.46 y7
815.45
912.53 y8
969.56 y9
970.63
1069.53
1070.63 y10
1071.56
1165.64
1183.66
1202.61
1203.79
1300.76 y12
1301.68
1442.74
1498.06
1613.85
1676.61
M/z
100 200 300 400 500 600 700 800 900 1000 1100 1200 1300 1400 1500 1600 1700

D

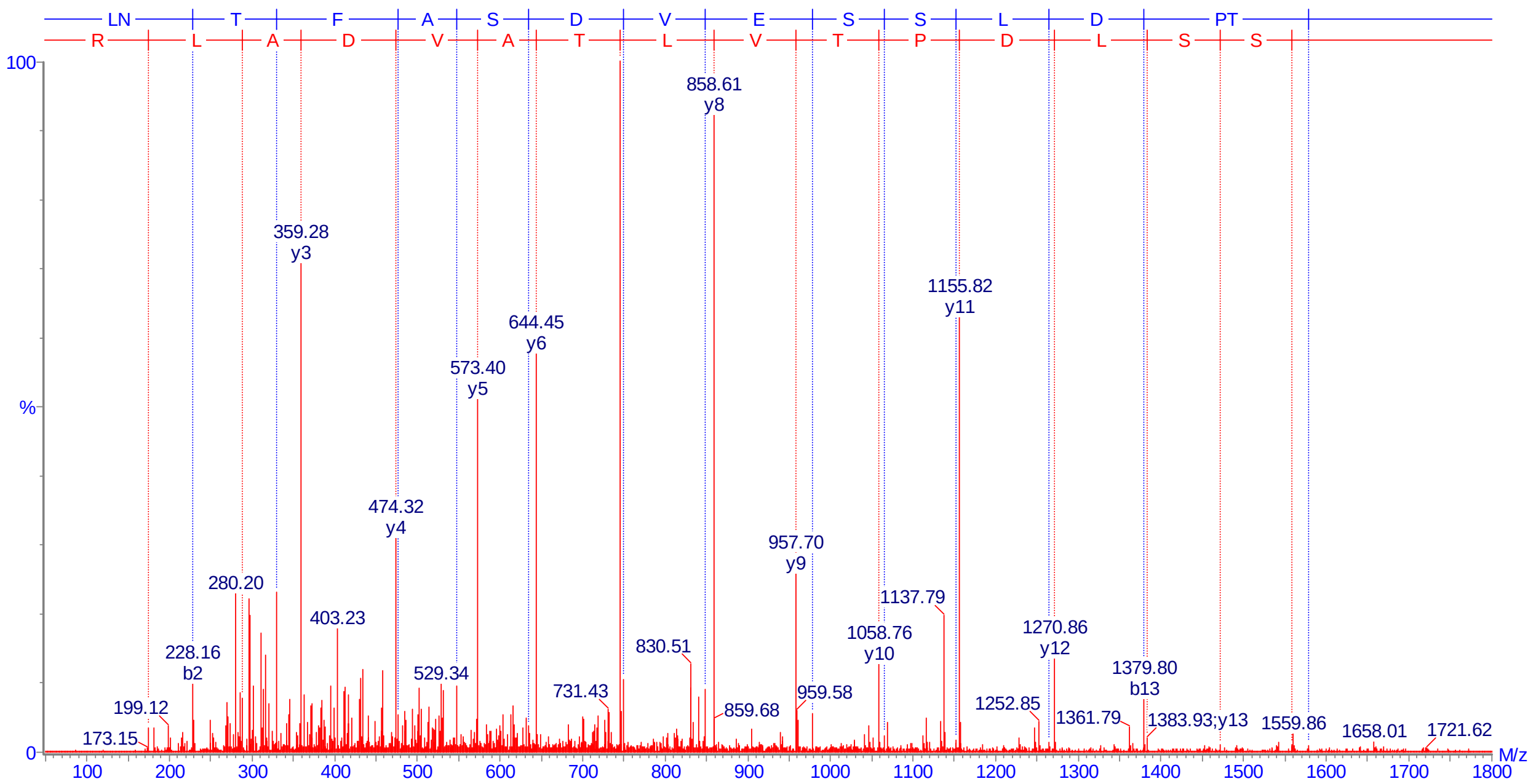
LN T F A S D V E S S L D PT
R L A D V A T L V T P D L S S
100
%
0
173.15
199.12
228.16 b2
280.20
359.28 y3
403.23
474.32 y4
529.34
573.40 y5
644.45 y6
731.43
830.51
858.61 y8
859.68
957.70 y9
959.58
1058.76 y10
1137.79
1155.82 y11
1252.85
1270.86 y12
1361.79
1379.80 b13
1383.93;y13
1559.86
1658.01
1721.62
M/z
100 200 300 400 500 600 700 800 900 1000 1100 1200 1300 1400 1500 1600 1700 1800
